# The dynamic changes and sex differences of 147 immune-related proteins during acute COVID-19 in 580 individuals

**DOI:** 10.1186/s12014-022-09371-z

**Published:** 2022-09-28

**Authors:** Guillaume Butler-Laporte, Edgar Gonzalez-Kozlova, Chen-Yang Su, Sirui Zhou, Tomoko Nakanishi, Elsa Brunet-Ratnasingham, David Morrison, Laetitia Laurent, Jonathan Afilalo, Marc Afilalo, Danielle Henry, Yiheng Chen, Julia Carrasco-Zanini, Yossi Farjoun, Maik Pietzner, Nofar Kimchi, Zaman Afrasiabi, Nardin Rezk, Meriem Bouab, Louis Petitjean, Charlotte Guzman, Xiaoqing Xue, Chris Tselios, Branka Vulesevic, Olumide Adeleye, Tala Abdullah, Noor Almamlouk, Yara Moussa, Chantal DeLuca, Naomi Duggan, Erwin Schurr, Nathalie Brassard, Madeleine Durand, Diane Marie Del Valle, Ryan Thompson, Mario A. Cedillo, Eric Schadt, Kai Nie, Nicole W. Simons, Konstantinos Mouskas, Nicolas Zaki, Manishkumar Patel, Hui Xie, Jocelyn Harris, Robert Marvin, Esther Cheng, Kevin Tuballes, Kimberly Argueta, Ieisha Scott, The Mount Sinai COVID-19 Biobank Team, Charuta Agashe, Priyal Agrawal, Alara Akyatan, Kasey Alesso-Carra, Eziwoma Alibo, Kelvin Alvarez, Angelo Amabile, Carmen Argmann, Kimberly Argueta, Steven Ascolillo, Rasheed Bailey, Craig Batchelor, Noam D Beckmann, Aviva G Beckmann, Priya Begani, Jessica Le Berichel, Dusan Bogunovic, Swaroop Bose, Cansu Cimen Bozkus, Paloma Bravo, Mark Buckup, Larissa Burka, Sharlene Calorossi, Lena Cambron, Guillermo Carbonell, Gina Carrara, Mario A. Cedillo, Christie Chang, Serena Chang, Alexander W. Charney, Steven T. Chen, Esther Cheng, Jonathan Chien, Mashkura Chowdhury, Jonathan Chung, Phillip H Comella, Dana Cosgrove, Francesca Cossarini, Liam Cotter, Arpit Dave, Travis Dawson, Bheesham Dayal, Diane Marie Del Valle, Maxime Dhainaut, Rebecca Dornfeld, Katie Dul, Melody Eaton, Nissan Eber, Cordelia Elaiho, Ethan Ellis, Frank Fabris, Jeremiah Faith, Dominique Falci, Susie Feng, Brian Fennessy, Marie Fernandes, Nataly Fishman, Nancy J. Francoeur, Sandeep Gangadharan, Daniel Geanon, Bruce D. Gelb, Benjamin S Glicksberg, Sacha Gnjatic, Joanna Grabowska, Gavin Gyimesi, Maha Hamdani, Diana Handler, Jocelyn Harris, Matthew Hartnett, Sandra Hatem, Manon Herbinet, Elva Herrera, Arielle Hochman, Gabriel E. Hoffman, Jaime Hook, Laila Horta, Etienne Humblin, Suraj Jaladanki, Hajra Jamal, Jessica S. Johnson, Gurpawan Kang, Neha Karekar, Subha Karim, Geoffrey Kelly, Jong Kim, Seunghee Kim-Schulze, Edgar Kozlova, Arvind Kumar, Jose Lacunza, Alona Lansky, Dannielle Lebovitch, Brian Lee, Grace Lee, Gyu Ho Lee, Jacky Lee, John Leech, Lauren Lepow, Michael B Leventhal, Lora E Liharska, Katherine Lindblad, Alexandra Livanos, Bojan Losic, Rosalie Machado, Kent Madrid, Zafar Mahmood, Kelcey Mar, Thomas U. Marron, Glenn Martin, Robert Marvin, Shrisha Maskey, Paul Matthews, Katherine Meckel, Saurabh Mehandru, Miriam Merad, Cynthia Mercedes, Elyze Merzier, Dara Meyer, Gurkan Mollaoglu, Sarah Morris, Konstantinos Mouskas, Emily Moya, Naa-akomaah Yeboah, Girish Nadkarni, Kai Nie, Marjorie Nisenholtz, George Ofori-Amanfo, Kenan Onel, Merouane Ounadjela, Manishkumar Patel, Vishwendra Patel, Cassandra Pruitt, Adeeb Rahman, Shivani Rathi, Jamie Redes, Ivan Reyes-Torres, Alcina Rodrigues, Alfonso Rodriguez, Vladimir Roudko, Panagiotis Roussos, Evelyn Ruiz, Pearl Scalzo, Eric E. Schadt, Ieisha Scott, Robert Sebra, Hardik Shah, Mark Shervey, Pedro Silva, Nicole W. Simons, Melissa Smith, Alessandra Soares-Schanoski, Juan Soto, Shwetha Hara Sridhar, Stacey-Ann Brown, Hiyab Stefanos, Meghan Straw, Robert Sweeney, Alexandra Tabachnikova, Collin Teague, Ryan Thompson, Manying Tin, Kevin Tuballes, Scott R. Tyler, Bhaskar Upadhyaya, Akhil Vaid, Verena Van Der Heide, Natalie Vaninov, Konstantinos Vlachos, Daniel Wacker, Laura Walker, Hadley Walsh, Wenhui Wang, Bo Wang, C. Matthias Wilk, Lillian Wilkins, Karen M. Wilson, Jessica Wilson, Hui Xie, Li Xue, Nancy Yi, Ying-chih Wang, Mahlet Yishak, Sabina Young, Alex Yu, Nina Zaks, Renyuan Zha, Celia M. T. Greenwood, Clare Paterson, Michael Hinterberg, Claudia Langenberg, Vincenzo Forgetta, Vincent Mooser, Thomas Marron, Noam Beckmann, Ephraim Kenigsberg, Alexander W. Charney, Seunghee Kim-schulze, Miriam Merad, Daniel E. Kaufmann, Sacha Gnjatic, J Brent Richards

**Affiliations:** 1 Lady Davis Institute, Jewish General Hospital, McGill University, Montréal, Québec Canada; 2 Department of Epidemiology, Biostatistics and Occupational Health, McGill University, Montréal, Québec Canada; 3 Department of Medicine, Icahn School of Medicine at Mount Sinai, New York, NY USA; 4 Department of Computer Science, McGill University, Montréal, Québec Canada; 5 Department of Human Genetics, McGill University, Montréal, Québec Canada; 6 Graduate School of Medicine, McGill International Collaborative School in Genomic Medicine, Kyoto University, KyotoKyoto, Japan; 7 Japan Society for the Promotion of Science, Tokyo, Japan; 8 Research Centre of the Centre Hospitalier de L’Université de Montréal, Montréal, Québec Canada; 9 Department of Emergency Medicine, Jewish General Hospital, McGill University, Montréal, Québec Canada; 10 MRC Epidemiology Unit, School of Clinical Medicine, University of Cambridge, Cambridge, UK; 11 Computational Medicine, Berlin Institute of Health at Charité—Universitätsmedizin Berlin, Berlin, Germany; 12 Infectious Diseases and Immunity in Global Health Program, Research Institute of the McGill University Health Centre, Montréal, Québec Canada; 13 Precision Immunology Institute, Icahn School of Medicine at Mount Sinai, New York, NY USA; 14 Genetics and Genomic Sciences, Icahn School of Medicine at Mount Sinai, New York, NY USA; 15 Department of Radiology, Icahn School of Medicine at Mount Sinai, New York, NY USA; 16 Human Immune Monitoring Center, Icahn School of Medicine at Mount Sinai, New York, NY USA; 17 SomaLogic Inc, Boulder, CO USA; 18 Early Phase Trials Unit, Mount Sinai Hospital, New York, NY USA; 19 Mount Sinai Clinical Intelligence Center, Icahn School of Medicine at Mount Sinai, New York, NY USA; 20 Department of Medicine, Université de Montréal, Montréal, Québec Canada; 21 Department of Twin Research, King’s College London, London, UK; 22 5 Prime Sciences, Montreal, Québec Canada; 23 McGill University, King’s College London (Honorary), Jewish General Hospital, Pavilion H-4133755 Côte-Ste-Catherine, Montréal, Québec H3T 1E2 Canada

**Keywords:** COVID-19, Proteomics, SOMAscan, Immunity

## Abstract

**Introduction:**

Severe COVID-19 leads to important changes in circulating immune-related proteins. To date it has been difficult to understand their temporal relationship and identify cytokines that are drivers of severe COVID-19 outcomes and underlie differences in outcomes between sexes. Here, we measured 147 immune-related proteins during acute COVID-19 to investigate these questions.

**Methods:**

We measured circulating protein abundances using the SOMAscan nucleic acid aptamer panel in two large independent hospital-based COVID-19 cohorts in Canada and the United States. We fit generalized additive models with cubic splines from the start of symptom onset to identify protein levels over the first 14 days of infection which were different between severe cases and controls, adjusting for age and sex. Severe cases were defined as individuals with COVID-19 requiring invasive or non-invasive mechanical respiratory support.

**Results:**

580 individuals were included in the analysis. Mean subject age was 64.3 (sd 18.1), and 47% were male. Of the 147 proteins, 69 showed a significant difference between cases and controls (p < 3.4 × 10^–4^). Three clusters were formed by 108 highly correlated proteins that replicated in both cohorts, making it difficult to determine which proteins have a true causal effect on severe COVID-19. Six proteins showed sex differences in levels over time, of which 3 were also associated with severe COVID-19: CCL26, IL1RL2, and IL3RA, providing insights to better understand the marked differences in outcomes by sex.

**Conclusions:**

Severe COVID-19 is associated with large changes in 69 immune-related proteins. Further, five proteins were associated with sex differences in outcomes. These results provide direct insights into immune-related proteins that are strongly influenced by severe COVID-19 infection.

**Supplementary Information:**

The online version contains supplementary material available at 10.1186/s12014-022-09371-z.

## Keypoints

- COVID-19 is associated with changes in cytokines, interleukins, and other immune-related proteins. However, previous research has failed to account for the dynamic nature of these changes over the course of infection, leading to often contradictory results.
- We measured 147 immune-related protein in 580 individuals in three large academic centers to precisely map the evolution of these proteins during acute COVID-19.
- COVID-19 was associated with a clear change in 69 proteins. More importantly, 3 of them may also help explain sex-differences in COVID-19 outcomes.
- These results provide greater insight into the COVID-19 immune response, and how it leads to severe illness.

**Supplementary Information:**

The online version contains supplementary material available at 10.1186/s12014-022-09371-z.

## Introduction

COVID-19 is characterized by a complex immune response which explains some of the observed variation in patient outcomes. In patients with a severe clinical course, some may develop a “COVID-19 cytokine storm” [1], though this term has been challenged due to a poor understanding of this response [2]. While previous publications found multiple cytokines and other immune-related proteins associated with COVID-19 outcomes [3–8], these associations were measured in small sample sizes, assessed a limited set of proteins, or did not provide a temporal analysis of the changes in cytokines during severe versus mild disease. These limitations may have contributed to contradicting results [9–11].

Similarly, it remains unclear if the host immune response explains a large proportion of differences in COVID-19 outcomes between males and females. While reports previously suggested that these differences were correlated with differential levels of cytokines (e.g. IL-8 and IL-18 [12]), these also suffered from small sample sizes and limited adjustment for temporal changes. These studies also likely contained many false positive associations due to multiple comparisons, as most of the sex-related differences were not replicated in other larger cohorts [13].

One way to address some of these limitations is by using high-throughput oligonucleotide-aptamer protein measurement technology [14]. These panels reliably measure thousands of blood circulating protein simultaneously, allowing for comprehensive measurements on larger number of subjects. The increase in sample size allows for better adjustments for time dependent changes in protein levels, providing a more granular understanding of their dynamics during infection. Here, we use the SOMAscan aptamer panel [15] (SomaLogic, Boulder, USA) in two prospectively enrolled cohorts from Canada and the United States (n = 580) to measure 147 proteins associated with the immune response over the first 14 days of COVID-19. This allowed us to clearly describe the temporal pattern of cytokines during COVID-19 disease progression.

By using large-scale protein measurement and accounting for temporal changes over the course of infection, we describe which proteins are likely associated with severe COVID-19, and which ones also underlie sex differences in outcomes.

## Methods

### Overview of study design

We used the SomaScan assay to measure 147 cytokines and other immune-related proteins in cases and controls in the Biobanque Québécoise de la COVID-19 [16] (BQC19) in Montreal, and in the Mount Sinai Biobank (MSB) at Icahn School of Medicine in New York City. We then combined those results using generalized additive models to identify proteins temporally associated with severe COVID-19.

### Population

The BQC19 and MSB are hospital-based prospective cohorts enrolling subjects with PCR proven SARS-CoV-2 infections, as well as individuals who presented with signs or symptoms consistent with COVID-19, but without a microbiological diagnosis of COVID-19. For this study, the BQC19 cohort was limited to subjects enrolled at the Jewish General Hospital and Centre Hospitalier de l’Université de Montréal, both university affiliated hospitals. Demographic characteristics and clinical risk factors were obtained by medical chart review or subject interview performed by clinicians or trained research coordinators in all cohorts. Specifically, time from onset of symptoms used for all analyses were recorded by trained clinical assistants or physicians based on medical records review or patient or relatives interview.

### COVID-19 case/control outcome definitions

Severe COVID-19 cases were defined as subjects with a positive SARS-CoV-2 PCR test result who either died or required invasive or non-invasive mechanical respiratory support. Mechanical respiratory support was defined as any one of the following: intubation, new positive airway pressure (CPAP) or bilevel positive airway pressure (BiPAP) ventilation, or high-flow nasal cannula. Controls were defined as any subjects with a positive PCR test who did not require invasive ventilation, or any subject with signs or symptoms consistent with COVID-19, but who had negative PCR tests for the virus. However, we also excluded participants who with severe non-COVID-19 disease (i.e. participants with respiratory support as defined above, but not due to COVID-19). This control definition was chosen to emphasize severe COVID-19 specific immune responses, as compared to a general hospital population.

### Protein measurements

We used the SOMAscan (v4) platform to measure 5284 circulating proteins from each participant, and then prioritized 147 immune-related proteins for the analysis. These proteins were selected to include all available interleukins (n = 38), CC motif chemokines (n = 23), CXC motif chemokines (n = 14), interferons (n = 17), toll-like receptors (n = 6), and immunoglobulins (n = 5) available from the SOMAscan panel, as well as 6 other proteins (G-CSF, GM-CSF, M-CSF, MIF, TNF-α, TNF-β) known to be involved in viral immune responses [17–19]. We also included all 38 soluble interleukin receptors measured by SOMAscan. These soluble receptors act as decoy receptors for their respective interleukins. Biologically, they bind to their interleukins in the circulation, preventing them from binding membrane-bound receptors, and having their usual biological effect. Their action may predict the effect of pharmacologic interleukin receptor blocking agents [20]. Owing to differences in the choice of aptamers in each SOMAscan panel, of the 147 proteins available in the BQC19 cohort, 15 were not available in the MSB cohort (IL-2, IL-7, IL-9, IL-34, IL-37, IL-12RB2, CCL1, CCL3, CXCL2, IFNB1, TLR2, IgD, IgE, IgG, and IgM). The full protein list in each cohort is available in Additional file 1.

To reflect acute illness, we limited this study to samples collected within 14 days of symptom onset (i.e. one sample per participant). To better control for the effect of COVID-19 treatment on circulating protein levels, we limited our analysis to only the first measurement of circulating proteins per subject, since these samples were less likely to be collected from individuals already starting therapy for severe COVID-19.

Samples were obtained and processed as per the manufacturer’s instructions. Briefly, blood samples were collected in acid-citrate-dextrose tubes (to prevent coagulation) and frozen at − 80 °C until analysis. Protein levels were measured using resonance fluorescence units, and further normalized and calibrated by SomaLogic to remove any systematic bias (e.g. batch effects). For the statistical analysis, we further standardized protein levels by subtracting their mean and dividing by their standard deviation to allow for easier interpretation and analysis.

### Statistical analysis

To find clusters of proteins that varied together, we first drew Spearman’s correlation heatmaps within cases and within controls separately. This was also done in both cohorts separately (i.e. 4 times in total). To better visualise Spearman correlation clusters, the proteins were ordered using a hierarchical clustering algorithm with the “complete linkage” method (implemented with the hclust base function in R [21], with default settings).

Second, to adjust for the time of onset of symptoms, which is expected to affect protein levels, we fit generalized additive models [22] (GAMs) on each protein levels during the first 14 days since symptom onset in cases and controls. In short, this analysis aims to model the natural history of protein levels by using measurements done at clinical presentation on different subjects (which have different time from onset of symptoms on presentation). GAMs fit spline between different immunity related proteins from days 1 to 14, it is therefore uniformly more powerful than dichotomizing protein levels in two time periods and comparing their levels. The GAMs were fit using cubic regression splines, the restricted maximum likelihood (REML) method, and with up to 15 knots allowed (the model chooses the optimal number of knots). All models were also adjusted for subjects’ age and sex. The GAMs obtained in the two cohorts were de-identified and meta-analyzed (if measured in both cohorts) using the metagam package [23] (v0.2.0). The resulting meta-analyzed models were then plotted for a 65-year-old male and female (65 was chosen because it was the mean age in the BQC19 cohort).

To check if the protein levels were different between cases and controls, we used GAM ANOVA using a model without case/control status as predictor of protein level as the nested model. Similarly, we used GAM ANOVA with nested models with and without sex variables to check for difference in cytokine levels between sexes. Approximate p-values for this null hypothesis of no difference between cases and controls were obtained using GAM ANOVA. GAMs were fitted using the mgcv package [22] (v1.8–33). Sample code is available in Additional file 2. Finally, GAM ANOVA p-values were meta-analyzed across cohorts using the logistic method with the metap package [24] (v1.4). We considered that protein levels differed between cases and controls if the resulting p-value was below Bonferroni correction (alpha = 0.05/147 = 0.0003). We acknowledge that this correction is overly conservative due to the correlatedness of protein levels.

All analyses were done using R [21] (v4.0.3).

**Table 1:** Subject characteristics in the two participating cohorts. Numbers presented as count (percentage) except where otherwise 570 noted. Hypertension information was not available for the Mount Sinai Biobank cohort.

	BQC19 (n = 333)		Mount Sinai Biobank (n = 247)	
	Cases (n = 91)	Controls (n = 242)	Cases (n = 119)	Controls (n = 128)
Age in years (mean)	67.2	66.2	64.8	59.2
Female sex	35 (38.5%)	133 (55.0%)	49 (41.2%)	57 (44.5%)
Hospital site	–	–	–	–
Centre Hospitalier de l’Université de Montréal	32 (35.2%)	22 (9.1%)	–	–
Jewish General Hospital	59 (64.8%)	220 (90.1%)	–	–
Mount Sinai Hospital	–	–		
COVID-19 positive	91 (100%)	202 (83.5%)	119 (100%)	128 (100%)
Diabetes	38 (41.8%)	71 (29.3%)	30 (25.2%)	32 (25.0%)
Chronic obstructive pulmonary disease	16 (17.6%)	27 (11.2%)	14 (11.8%)	8 (6.3%)
Chronic kidney disease	16 (17.6%)	24 (9.9%)	15 (12.6%)	26 (20.3%)
Congestive heart failure	13 (14.3%)	28 (11.6%)	14 (11.8%)	11 (8.6%)
Hypertension	60 (65.9%)	134 (55.4%)	–	–
Liver disease	2 (2.2%)	4 (1.7%)	6 (5.0%)	4 (3.1%)
Smoking status				
Current smoker	5 (5.5%)	6 (2.5%)	10 (8.4%)	9 (7.0%)
Ex-smoker	11 (12.1%)	30 (12.4%)	32 (26.9%)	35 (27.3%)
Never smoker	39 (42.9%)	172 (71.1%)	47 (39.5%)	65 (50.8)
Don’t know	36 (39.6%)	34 (14.0%)	30 (25.2%)	19 (14.8%)

## Results

### Population

Table 1 shows basic characteristic of the participants in each cohort. Mean age was similar between cases and controls in the BQC19 (67.2 vs 66.2 year-old), but cases were slightly older in the MSB (64.8 vs 59.2 year-old). In both cohort, there were less females amongst cases compared to controls: 38.5% vs 55.0% in the BQC19, and 41.2% vs 44.5% in the MSB. There were more diabetic cases than controls in the BQC19 (41.8% vs 29.3%) but a similar proportion in the MSB. In both cohorts, there were more cases with chronic obstructive pulmonary disease: 17.6% vs 11.2% in the BQC-19, and 11.8% vs 6.3% in the MSB. There were also more heart failure diagnoses in cases in both cohorts: 14.3% vs 11.6% in the BQC-19, and 11.8% vs 8.6% in the MSB. Finally, there were less never-smokers amongst cases: 42.9% vs 71.1% in the BQC-19, and 39.5% vs 50.8% in the MSB. These values are comparable to other reported large COVID-19 cohorts [25].

### Immune-related protein levels dynamics over time

Many cytokines and related proteins showed statistically significant time-dependent differences between cases and controls (Bonferroni threshold 0.05/147 = 0.00034): 17 of the 38 interleukins, 24 of the 38 soluble interleukin receptors, 11 of the 23 CC chemokines, 6 of the 14 CXC chemokines, 8 of the 17 interferons related proteins, and 3 of 17 other immune-related proteins (Table 2).

**Table 2:** Immune-related proteins with differences between severe COVID-19 cases and controls in our meta-analysis of the BQC-19 and MSB results (Bonferroni adjusted threshold 0.05/147 = 0.00034)

Proteins	P-values	Proteins	P-values
**Interleukins**		**Soluble interleukin receptors**	
IL1A	9.04 × 10^–6^	IL1R1	1.50 × 10^–8^
IL1B	1.21 × 10^–6^	IL1R2	1.91 × 10^–7^
IL3	2.34 × 10^–4^	IL1RAPL2	4.23 × 10^–11^
IL4	1.35 × 10^–11^	IL1RL1	2.90 × 10^–12^
IL6	9.50 × 10^–10^	IL1RL2	1.45 × 10^–4^
IL11	1.14 × 10^–10^	IL1RN	2.89 × 10^–6^
IL12	1.26 × 10^–4^	IL2RB	1.04 × 10^–4^
IL13	3.04 × 10^–7^	IL3RA	4.37 × 10^–7^
IL17D	1.66 × 10^–8^	IL4R	1.67 × 10^–8^
IL17F	2.14 × 10^–10^	IL7R	5.12 × 10^–11^
IL18	8.26 × 10^–7^	^b^IL10RB	6.18 × 10^–5^
IL19	2.32 × 10^–4^	IL10RA.soma2	5.17 × 10^–8^
IL24	1.52 × 10^–11^	IL11RA	1.64 × 10^–6^
IL25	2.76 × 10^–7^	IL12RB1	5.46 × 10^–13^
IL36B	1.52 × 10^–9^	IL13RA1	5.15 × 10^–7^
IL36G	1.28 × 10^–6^	^b^IL15RA.soma2	1.10 × 10^–7^
^a^IL37	3.17 × 10^–4^	IL17RB	1.22 × 10^–4^
**Interferons**		IL17RC	1.02 × 10^–4^
IFNA4	9.47 × 10^–8^	IL18RAP	1.50 × 10^–5^
IFNA6	1.09 × 10^–7^	IL21R	1.28 × 10^–11^
IFNA8	2.43 × 10^–5^	IL22RA1	1.66 × 10^–14^
IFNA10	4.74 × 10^–4^	IL22RA2	1.10 × 10^–5^
^a^IFNB1	1.80 × 10^–4^	IL23R	5.80 × 10^–5^
IFNL1	6.53 × 10^–5^	IL27RA	2.11 × 10^–6^
IFNL2	3.01 × 10^–11^	**CXCL chemokines**	
IFNL3	3.60 × 10^–5^	CXCL5	4.53 × 10^–8^
**CCL chemokines**		CXCL10	3.22 × 10^–9^
CCL7	5.21 × 10^–10^	CXCL12	1.21 × 10^–4^
CCL8	4.14 × 10^–5^	CXCL13	1.01 × 10^–5^
CCL11	5.23 × 10^–6^	CXCL14	5.42 × 10^–10^
CCL13	4.01 × 10^–11^	CXCL16	2.26 × 10^–5^
CCL19	1.93 × 10^–6^	**Others**	
CCL20	1.30 × 10^–6^	M-CSF	1.60 × 10^–13^
CCL22	8.84 × 10^–10^	TLR1.soma1	2.62 × 10^–4^
CCL23	8.15 × 10^–6^	LT-α / TNF-β	2.08 × 10^–10^
CCL24	3.50 × 10^–10^		
CCL26	2.78 × 10^–8^		
CCL27	3.27 × 10^–13^		

^a^For IL37 and IFNB1, the p-values from the BQC-19 p-value are shown (protein not available in the MSB panel)

^b^For IL10RB and IL15RA.soma2, the p-value from the MSB p-value is shown (GAM ANOVA approximation failure)

**Fig. 1:**
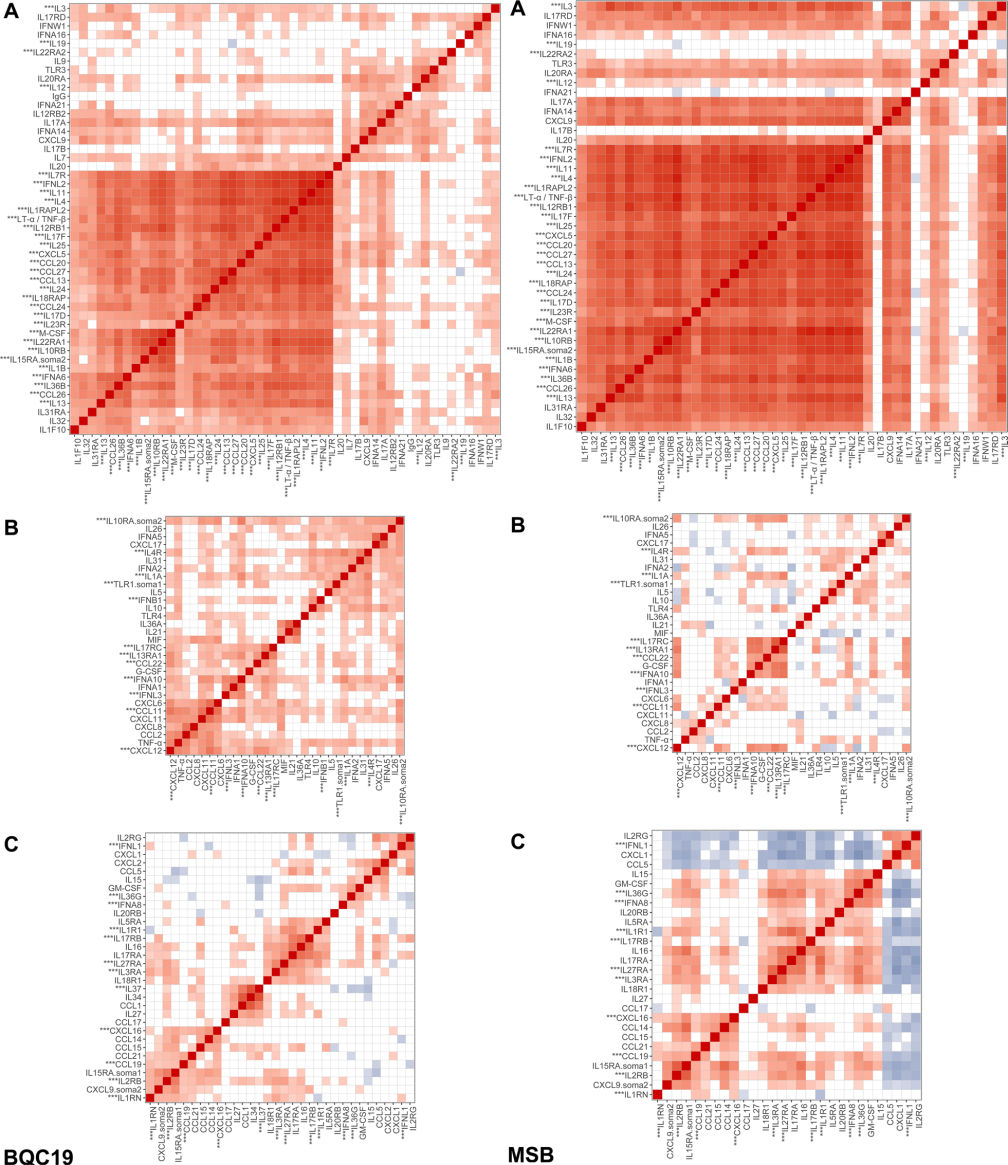
Spearman correlations for three clusters (A, B and C) of proteins in the BQC (left) and the MSB (right). Only correlations with p-values less than 0.05 shown. Proteins with asterisks (***) showed a statistically significant differences between cases and controls (Bonferroni threshold 0.05/147). Full spearman correlation heatmap available in Additional file 3

Hierarchical clustering and Spearman correlation delineated clear clusters of proteins that varied together over the course of infection (Fig. 1 and Additional file 3). Visual inspection of the BQC19 with the MSB heatmaps reveals three large protein clusters whose members show similar changes in levels. Importantly, the cluster with the highest proportion number of proteins showing an association with case and control status (cluster A; 31 out of 49 proteins) is also the one with the highest mean absolute Spearman correlation in both cohorts. Cluster A also showed a clear increase in Spearman correlations between cases and controls (mean increase of 0.163 across cohorts), supporting the fact that an immune overactivation underlies severe COVID-19 (Additional file 3). Proteins in this cluster show increasing levels in cases over the first 14 days of symptoms, whereas they remain stable in controls. In contrast, clusters B and C showed a negative correlation with cluster A, with higher but slowly tapering protein levels in controls. These clusters did not replicate as clearly between cohorts and have a lower proportion of proteins associated with case and control status.

**Fig. 2:**
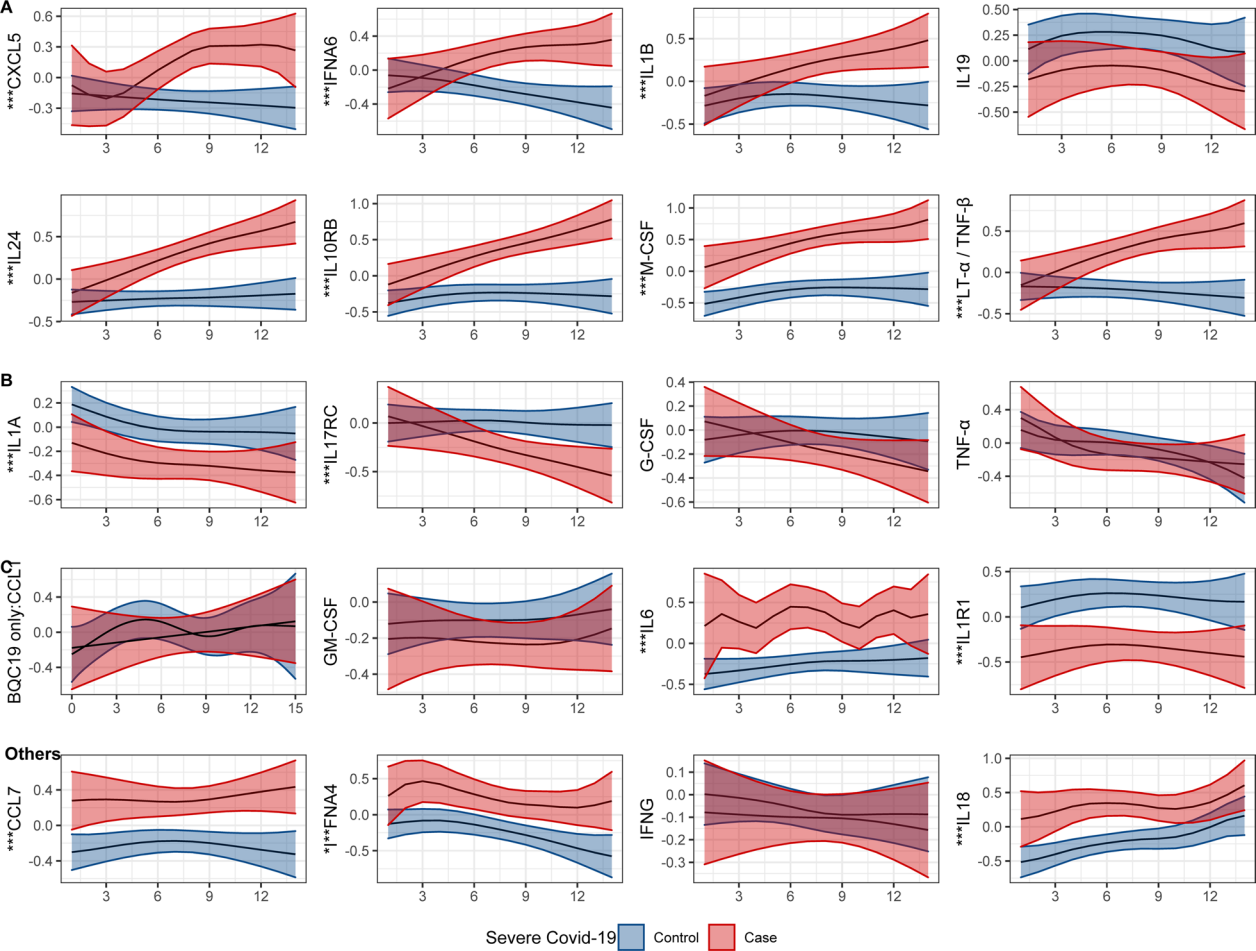
Smoothed curves for cluster-representative immune-related proteins, as a function of days since symptoms onset (x-axis), and separately for severe COVID cases and controls. Estimated curves are shown for 65-year-old. Y-axis is standardized to a mean of 0 and standard deviation of 1. Full results are shown in Additional final 5. Blue: controls. Red: severe COVID-19. Asterisks (***): p < 3.4 × 10^–4^ for case–control difference in protein levels

Each cluster contained a heterogeneous set of proteins (Additional file 4), with many replicating previously published findings [3–7], supporting the robustness of our results. Of note, many of the interleukin-10 family cytokines (IL19, IL20, IL24) or their soluble receptors (IL10RB, IL20A, IL22RA1 and IL22RA2) were present in cluster A. Other notable proteins found in cluster A include two members of the IL1 family (IL1B and IL36B), IL11 (a member of the IL6 family which both act on the same receptor [26]), multiple members of the IL17 family (IL17A, IL17B, IL17D, IL17F, and IL25), and IL4 and IL13 which both act on the same receptor to drive severe asthma [27]. Interestingly, clusters B and C contained some proteins related to those in cluster A which still showed significant differences between cases and controls. These included IL1A, IL4R (the soluble receptor for IL4), and IL10RA (a component of the soluble receptor for IL10) in cluster B, and IL1R1 (a soluble receptor for IL1) in cluster C. Figure 2 shows some representative proteins from each cluster and from the rest of the proteins, and all protein level plots are shown in Additional file 5.

### Differences in cytokine levels between sexes in cases and controls over time

**Fig. 3:**
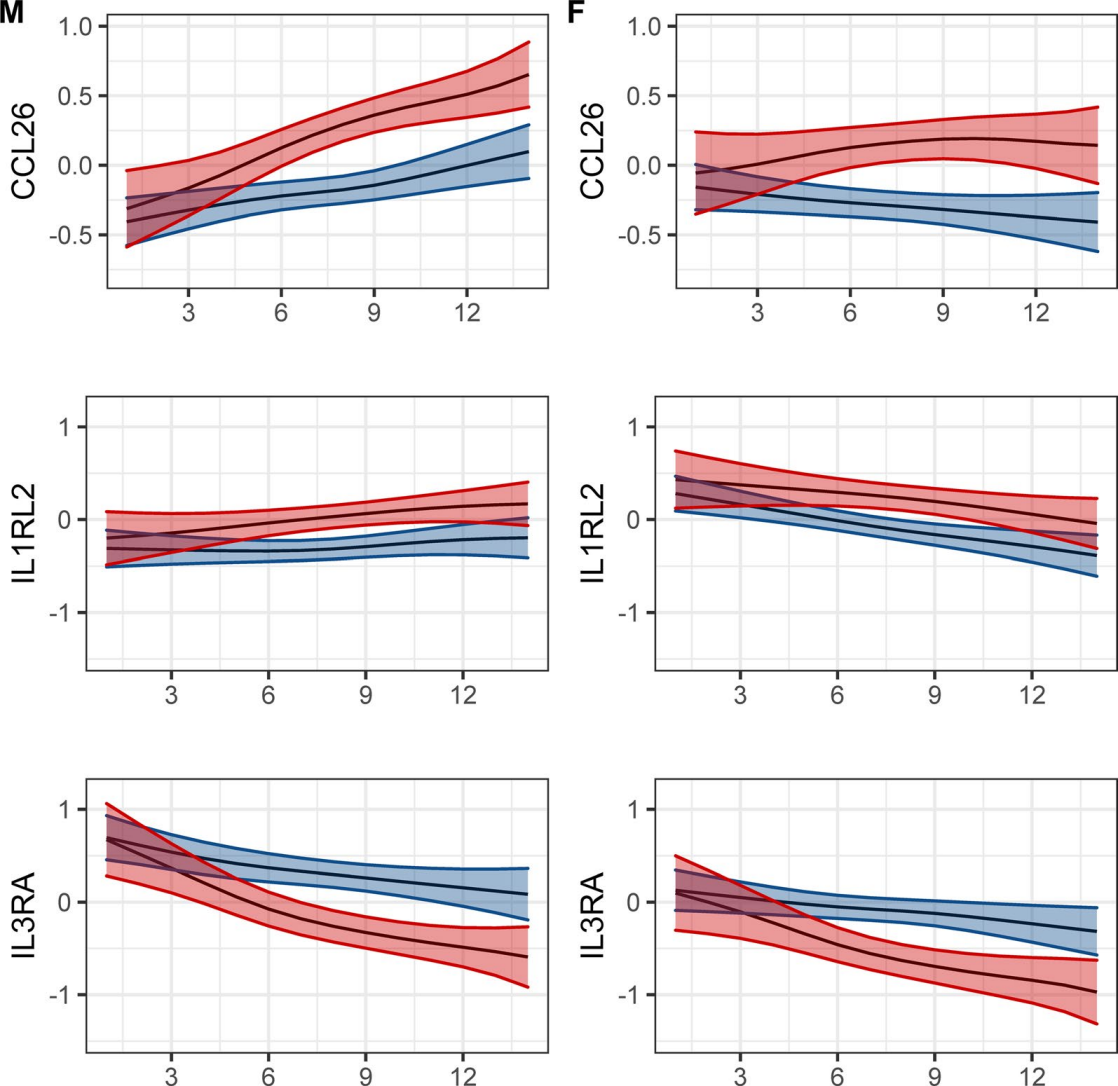
Smoothed protein level curves showing time-related and sex-related differences as a function of days since symptoms onset (x-axis) in a 65-year-old patient (p < 3.4 × 10^–4^ for sex differences in cytokine levels). Y-axis is standardized to a mean of 0 and standard deviation of 1 F: female. M: male. Blue: controls. Red: severe COVID-19

Of the 147 proteins, 5 showed a difference between males and females: TLR5, CXCL17, CCL28, CCL26, IL1RL2, and IL3RA. However, only the last three were also associated with case and control status (Fig. 3). CCL6 was found in the previously described cluster A of highly associated proteins, while IL3A was in cluster C. Both proteins had slightly different levels on the first day of symptoms (higher in females for CCL26, lower in females for IL3RA) but trended similarly afterwards. ILRL2 did not cluster well with other proteins and decreased towards normal levels more rapidly in females. Of note, IL3RA is the only one of these proteins for which the corresponding genes is located on a sex chromosome (chromosome X).

## Discussion

In this study, we used two large prospective cohorts and a panel measuring 147 circulating immune-related proteins and found that severe COVID-19 was associated with a clear activation in many immune-related proteins, with most protein levels varying together closely overt time. These results also provided three proteins that were importantly different between the sexes: CCL26, IL1RL2 and IL3RA. These 3 sex-specific protein findings were not found in previous reports, which is partly explained by the fact that our panel included more proteins than other studies, but this could also suggest false positive associations due to multiple testing. Hence, while most of the changes in immune-related proteins observed in severe COVID-19 are shared across sexes, it remains possible that disparity in outcomes between sexes may be mediated by differences in immune-related proteins levels.

Our study’s main strengths include its large sample size with strong replication between the two independent cohorts, the large protein measurement panel and the fact that proteins were measured at different times during infection, a feature that was explicitly modelled into our analysis. These provided a granular depiction of time-dependent immune responses to COVID-19 and explain previously discordant reports on the association between different immune proteins and COVID-19. For example, visual inspection of the interferon level dynamics will often reveal that the differences in measurement timing can easily explain previously reported differences in direction of associations [11]. Our large sample and careful adjustments for multiple comparisons also likely avoided spurious associations.

Other studies have assessed at protein levels in acute COVID-19, and we share many of the same observations already made. For example, severe COVID-19 was linked to changes in IL-13 [3], IL6 and IL-1B [4], and multiple chemokines (e.g. CCL20, CCL27, CXCL10 [28]). Hence, while it is clear that immune-related proteins levels are associated with outcomes, differences in methodology led to varying observations. For example, Lucas et al [3] observed differences in IL-1B, IL-6, IL-18, and TNF-α between severe and non-severe individuals using the Eve Technologies (Calgary, Alberta, Canada) Luminex based HD71 assay. However, using a different Luminex-based assay, Wilson et al [29] found no such difference in these 4 cytokines between severe COVID-19 cases and non-COVID-19 sepsis controls. Different results were again obtained from Filbin et al [30] who used the Olink (Uppsala, Sweden) multiplex antibody-oligonucleotide assay to highlight IL6, IL-1RL1, and IL-1RN’s role in severe COVID-19. As mentioned above, these differences can likely be explained by either small sample sizes, insufficient control for time of onset of symptoms, and different choices of cases and controls. Indeed, we replicated the IL6, IL-1RL1, and IL-1RN results from Filbin et al. which is to our knowledge the previously largest proteomics study on acute Covid-19. This study used similar methods to ours but adjusted for day of hospitalization rather than onset of symptoms. Comparisons to other studies should therefore also keep these methodological differences in mind.

Less is known about the role of immune-related proteins in COVID-19 outcome sex differences. A previous report [12] suggested a role for IL8 and IL18, but these were not replicated in other studies [13]. Our study is the first to report on difference in TLR5, CXCL17, CCL26, IL1RL2, or IL3RA levels in sexes during infection. While the mechanism by which they could influence outcomes is unclear, it is worth noting that the gene encoding for IL3RA is located on the X chromosome, providing a plausible explanation for the observed difference. Further CCL26 (also known as eotaxin-3) is known to induce eosinophils tissue infiltration [31], which could influence COVID-19 outcomes [32]. However, multiple studies have shown differences in cellular immune responses, COVID-19 specific antibody levels, and many commonly measured inflammatory markers in clinical practice (e.g. C-reactive protein) [12, 33]. Hence, it remains possible that another immune pathway that was not measured by our panel might be involved in the observed sex differences in outcomes. However, our observations on TLR5, CXCL17, CCL26, IL1RL2, and IL3RA provide clear proteins to explore to explain sex differences in COVID-19 outcomes.

Nevertheless, our study still has limitations. First, while we assayed proteins in the first collected samples, it remains possible that some subjects received immunomodulatory drugs (e.g. dexamethasone) which would have affected protein levels. However, this would likely attenuate the differences between the cases and the controls, and our results would therefore be biased towards the null hypothesis. Second, given that protein time trends were obtained using multiple different subjects, unmeasured confounders could explain some of our findings. While these cannot be easily measured, it is reassuring that our results replicated across two cohorts, arguing against the presence of confounders with large effect sizes. Third, the control group made up of non-severe COVID-19 participants as well as non-COVID-19 disease may have biased some of our results towards the null. However, this specific choice of control arm made our results more specific for severe COVID-19, rather than critical illness in general, and we still found clear associations with many immune-related proteins. Fourth, the use of SOMAscan may make comparisons difficult with other studies using different protein measurement technologies. Despite this, SOMAscan showed great sensitivity, specificity, and reproducibility when benchmarked against mass spectrometry [34], and our conclusions are unlikely to be greatly biased by the choice of protein measuring platform. Lastly, while this is one of the largest panels of immune-related proteins studied for COVID-19, there are multiple proteins that were not measured, and we cannot assess whether other unmeasured proteins may also have important effects on the outcomes.

In conclusion, using two large independent cohorts with broad protein measurements, we showed that severe COVID-19 was associated with clear time-dependent changes in multiple immune-related proteins, and that these may in part explain difference in COVID-19 outcomes between sexes.

## Supplementary Information

**Additional file 1: **List of immunity-related proteins measured.

**Additional file 2: **Sample code for the generalized additive models.

**Additional file 3: **Protein correlation heatmaps.

**Additional file 4: **Protein clusters.

**Additional file 5: **Inferred protein levels over time.

**Additional file 6: **Mount Sinai investigators.

## Data Availability

The BQC19 is an Open Science Biobank. Instructions on how to access data for individuals from the BQC19 at the Jewish General Hospital site is available at https://www.mcgill.ca/genepi/mcg-covid-19-biobank. Instructions on how to access data from other sites of the BQC19 is available at https://www.bqc19.ca/en/access-data-samples.
