# Correction: The dynamic changes and sex differences of 147 immune-related proteins during acute COVID-19 in 580 individuals

**DOI:** 10.1186/s12014-022-09378-6

**Published:** 2022-11-15

**Authors:** Guillaume Butler-Laporte, Edgar Gonzalez-Kozlova, Chen-Yang Su, Sirui Zhou, Tomoko Nakanishi, Elsa Brunet-Ratnasingham, David Morrison, Laetitia Laurent, Jonathan Aflalo, Marc Aflalo, Danielle Henry, Yiheng Chen, Julia Carrasco-Zanini, Yossi Farjoun, Maik Pietzner, Nofar Kimchi, Zaman Afrasiabi, Nardin Rezk, Meriem Bouab, Louis Petitjean, Charlotte Guzman, Xiaoqing Xue, Chris Tselios, Branka Vulesevic, Olumide Adeleye, Tala Abdullah, Noor Almamlouk, Yara Moussa, Chantal DeLuca, Naomi Duggan, Erwin Schurr, Nathalie Brassard, Madeleine Durand, Diane Marie Del Valle, Ryan Thompson, Mario A. Cedillo, Eric Schadt, Kai Nie, Nicole W. Simons, Konstantinos Mouskas, Nicolas Zaki, Manishkumar Patel, Hui Xie, Jocelyn Harris, Robert Marvin, Esther Cheng, Kevin Tuballes, Kimberly Argueta, Ieisha Scott, The Mount Sinai COVID-19 Biobank Team, Celia M. T. Greenwood, Clare Paterson, Michael Hinterberg, Claudia Langenberg, Vincenzo Forgetta, Vincent Mooser, Thomas Marron, Noam D. Beckmann, Ephraim Kenigsberg, Alexander W. Charney, Seunghee Kim-schulze, Miriam Merad, Daniel E. Kaufmann, Sacha Gnjatic, J. Brent Richards

**Affiliations:** 1 Lady Davis Institute, Jewish General Hospital, McGill University, Montreal, QC Canada; 2 Department of Epidemiology, Biostatistics and Occupational Health, McGill University, Montreal, QC Canada; 3 Department of Medicine, Icahn School of Medicine at Mount Sinai, New York, NY USA; 4 Department of Computer Science, McGill University, Montreal, QC Canada; 5 Department of Human Genetics, McGill University, Montreal, QC Canada; 6 Graduate School of Medicine, McGill International Collaborative School in Genomic Medicine, Kyoto University, Kyoto, Kyoto Japan; 7 Japan Society for the Promotion of Science, Tokyo, Japan; 8 Research Centre of the Centre Hospitalier de L’Université de Montréal, Montreal, QC Canada; 9 Department of Emergency Medicine, Jewish General Hospital, McGill University, Montreal, QC Canada; 10 MRC Epidemiology Unit, School of Clinical Medicine, University of Cambridge, Cambridge, UK; 11 Computational Medicine, Berlin Institute of Health at Charité—Universitätsmedizin Berlin, Berlin, Germany; 12 Infectious Diseases and Immunity in Global Health Program, Research Institute of the McGill University Health Centre, Montreal, QC Canada; 13 Precision Immunology Institute, Icahn School of Medicine at Mount Sinai, New York, NY USA; 14 Genetics and Genomic Sciences, Icahn School of Medicine at Mount Sinai, New York, NY USA; 15 Department of Radiology, Icahn School of Medicine at Mount Sinai, New York, NY USA; 16 Human Immune Monitoring Center, Icahn School of Medicine at Mount Sinai, New York, NY USA; 17 SomaLogic Inc, Boulder, CO USA; 18 Early Phase Trials Unit, Mount Sinai Hospital, New York, NY USA; 19 Mount Sinai Clinical Intelligence Center, Icahn School of Medicine at Mount Sinai, New York, NY USA; 20 Department of Medicine, Université de Montréal, Montreal, QC Canada; 21 Department of Twin Research, King’s College London, London, UK; 22 5 Prime Sciences, Montreal, QC Canada; 23 McGill University, King’s College London (Honorary), Jewish General Hospital, Pavilion H-4133755 Côte-Ste-Catherine, Montreal, QC H3T 1E2 Canada

## Correction: Clinical Proteomics (2022) 19:34 10.1186/s12014-022-09371-z

In the original publication of the article [1], the author name Noam D. Beckmann was incorrectly written as Noam Beckmann. The original article has been corrected.
