# Supplementary material for: The dynamic changes and sex differences of 147 immune-related proteins during acute COVID-19 in 580 individuals
Source: Clin Proteomics. 2022 Sep 28;19:34. doi: 10.1186/s12014-022-09371-z (PMC9516500; doi:10.1186/s12014-022-09371-z)
Supplement: Supplementary file 1 — Additional file 1: List of immunity-related proteins measured. [file 12014_2022_9371_MOESM1_ESM.docx]

**Supplement 1**: Immune-related protein list

BQC19: Biobanque Québécoise de la Covid-19

MSB: Mount Sinai Biobank

| **Interleukins** | | | |
| --- | --- | --- | --- |
| **SOMAscan identifier** | **Protein Name** | **In BQC19?** | **In MSB?** |
| IL1A.4851.25 | IL1A | Yes | Yes |
| IL1B.3037.62 | IL1B | Yes | Yes |
| IL1F10.17356.34 | IL1F10 | Yes | Yes |
| IL2.3070.1 | IL2 | Yes | No |
| IL3.4717.55 | IL3 | Yes | Yes |
| IL4.2906.55 | IL4 | Yes | Yes |
| IL5.11071.1 | IL5 | Yes | Yes |
| IL6.4673.13 | IL6 | Yes | Yes |
| IL7.4140.3 | IL7 | Yes | No |
| IL9.5834.18 | IL9 | Yes | No |
| IL10.2773.50 | IL10 | Yes | Yes |
| IL11.4493.92 | IL11 | Yes | Yes |
| IL12A.IL12B.10367.62 | IL12 | Yes | Yes |
| IL13.3072.4 | IL13 | Yes | Yes |
| IL15.19568.17 | IL15 | Yes | Yes |
| IL16.2774.10 | IL16 | Yes | Yes |
| IL17A.9170.24 | IL17A | Yes | Yes |
| IL17B.3499.77 | IL17B | Yes | Yes |
| IL17C.9255.5 | IL17C | Yes | Yes |
| IL17D.4136.40 | IL17D | Yes | Yes |
| IL17F.14026.24 | IL17F | Yes | Yes |
| IL18.5661.15 | IL18 | Yes | Yes |
| IL19.3035.80 | IL19 | Yes | Yes |
| IL20.4138.25 | IL20 | Yes | Yes |
| IL21.7124.18 | IL21 | Yes | Yes |
| IL22.2778.10 | IL22 | Yes | Yes |
| IL12B.IL23A.10365.132 | IL23 | Yes | Yes |
| IL24.3321.2 | IL24 | Yes | Yes |
| IL25.4137.57 | IL25 | Yes | Yes |
| IL26.16760.2 | IL26 | Yes | Yes |
| IL27.EBI3.2829.19 | IL27 | Yes | Yes |
| IL31.10455.196 | IL31 | Yes | Yes |
| IL32.9051.13 | IL32 | Yes | Yes |
| IL34.4556.10 | IL34 | Yes | No |
| IL36A.14150.7 | IL36A | Yes | Yes |
| IL36B.14149.9 | IL36B | Yes | Yes |
| IL36G.9117.4 | IL36G | Yes | Yes |
| IL37.2723.9 | IL37 | Yes | No |

| **CC chemokines** | | | |
| --- | --- | --- | --- |
| **SOMAscan identifier** | **Protein Name** | **In BQC19?** | **In MSB?** |
| CCL1.2770.51 | CCL1 | Yes | No |
| CCL2.2578.67 | CCL2 | Yes | Yes |
| CCL3.3040.59 | CCL3 | Yes | No |
| CCL5.5480.49 | CCL5 | Yes | Yes |
| CCL7.4886.3 | CCL7 | Yes | Yes |
| CCL8.13748.4 | CCL8 | Yes | Yes |
| CCL11.5301.7 | CCL11 | Yes | Yes |
| CCL13.4144.13 | CCL13 | Yes | Yes |
| CCL14.2900.53 | CCL14 | Yes | Yes |
| CCL15.18289.16 | CCL15 | Yes | Yes |
| CCL16.4913.78 | CCL16 | Yes | Yes |
| CCL17.3519.3 | CCL17 | Yes | Yes |
| CCL18.3044.3 | CCL18 | Yes | Yes |
| CCL19.4922.13 | CCL19 | Yes | Yes |
| CCL20.2468.62 | CCL20 | Yes | Yes |
| CCL21.2516.57 | CCL21 | Yes | Yes |
| CCL22.3508.78 | CCL22 | Yes | Yes |
| CCL23.2913.1 | CCL23 | Yes | Yes |
| CCL24.4128.27 | CCL24 | Yes | Yes |
| CCL25.2705.5 | CCL25 | Yes | Yes |
| CCL26.9168.31 | CCL26 | Yes | Yes |
| CCL27.2192.63 | CCL27 | Yes | Yes |
| CCL28.2890.59 | CCL28 | Yes | Yes |

| **CXC chemokines** | | | |
| --- | --- | --- | --- |
| **SOMAscan identifier** | **Protein Name** | **In BQC19?** | **In MSB?** |
| CXCL1.2985.35 | CXCL1 | Yes | Yes |
| CXCL2.3148.49 | CXCL2 | Yes | No |
| CXCL5.2979.8 | CXCL5 | Yes | Yes |
| CXCL6.3495.15 | CXCL6 | Yes | Yes |
| CXCL8.3447.64 | CXCL8 | Yes | Yes |
| CXCL9.11593.21 | CXCL9 | Yes | Yes |
| CXCL9.9188.119 | CXCL9.soma2 | Yes | Yes |
| CXCL10.4141.79 | CXCL10 | Yes | Yes |
| CXCL11.3038.9 | CXCL11 | Yes | Yes |
| CXCL12.3516.60 | CXCL12 | Yes | Yes |
| CXCL13.3487.32 | CXCL13 | Yes | Yes |
| CXCL14.5730.60 | CXCL14 | Yes | Yes |
| CXCL16.2436.49 | CXCL16 | Yes | Yes |
| CXCL17.9495.10 | CXCL17 | Yes | Yes |

| **Interferons** | | | |
| --- | --- | --- | --- |
| **SOMAscan identifier** | **Protein Name** | **In BQC19?** | **In MSB?** |
| IFNA1.18389.11 | IFNA1 | Yes | Yes |
| IFNA2.3497.13 | IFNA2 | Yes | Yes |
| IFNA4.15405.23 | IFNA4 | Yes | Yes |
| IFNA5.6210.100 | IFNA5 | Yes | Yes |
| IFNA6.5714.88 | IFNA6 | Yes | Yes |
| IFNA7.14129.1 | IFNA7 | Yes | Yes |
| IFNA8.6214.84 | IFNA8 | Yes | Yes |
| IFNA10.14128.121 | IFNA10 | Yes | Yes |
| IFNA14.7180.114 | IFNA14 | Yes | Yes |
| IFNA16.6421.52 | IFNA16 | Yes | Yes |
| IFNA21.15404.3 | IFNA21 | Yes | Yes |
| IFNB1.14127.240 | IFNB1 | Yes | No |
| IFNG.15346.31 | IFNG | Yes | Yes |
| IFNL1.4396.54 | IFNL1 | Yes | Yes |
| IFNL2.4397.26 | IFNL2 | Yes | Yes |
| IFNL3.5713.9 | IFNL3 | Yes | Yes |
| IFNW1.7196.21 | IFNW1 | Yes | Yes |

| **Other immune related proteins** | | | |
| --- | --- | --- | --- |
| **SOMAscan identifier** | **Protein Name** | **In BQC19?** | **In MSB?** |
| CSF3.4840.73 | G-CSF | Yes | Yes |
| CSF2.4697.59 | GM-CSF | Yes | Yes |
| CSF1.3738.54 | M-CSF | Yes | Yes |
| MIF.8221.19 | MIF | Yes | Yes |
| TNF.5936.53 | TNF-α | Yes | Yes |
| LTA.4703.87 | LT-α / TNF-β | Yes | Yes |
| TLR1.11149.3 | TLR1.soma1 | Yes | Yes |
| TLR1.16324.38 | TLR1.soma2 | Yes | Yes |
| TLR2.3835.11 | TLR2 | Yes | No |
| TLR3.16918.198 | TLR3 | Yes | Yes |
| TLR4.11101.18 | TLR4 | Yes | Yes |
| TLR5.18935.14 | TLR5 | Yes | Yes |
| IGHA1.IGHA2.11089.7 | IgA | Yes | Yes |
| IGHD.IGK.IGL.4916.2 | IgD | Yes | No |
| IGHE.IGK.IGL.4135.84 | IgE | Yes | No |
| IGHG1.IGHG2.IGHG3.IGHG4.IGK.IGL.2744.57 | IgG | Yes | No |
| IGHM.IGJ.IGK.IGL.3069.52 | IgM | Yes | No |

| **Soluble interleukin receptors** | | | |
| --- | --- | --- | --- |
| **SOMAscan identifier** | **Protein Name** | **In BQC19?** | **In MSB?** |
| IL1R1.2991.9 | IL1R1 | Yes | Yes |
| IL1R2.14133.93 | IL1R2 | Yes | Yes |
| IL1RAP.14048.7 | IL1RAP | Yes | Yes |
| IL1RAPL2.5082.51 | IL1RAPL2 | Yes | Yes |
| IL1RL1.4234.8 | IL1RL1 | Yes | Yes |
| IL1RL2.2994.71 | IL1RL2 | Yes | Yes |
| IL1RN.5353.89 | IL1RN | Yes | Yes |
| IL2RA.3151.6 | IL2RA | Yes | Yes |
| IL2RB.9343.16 | IL2RB | Yes | No |
| IL2RG.2634.2 | IL2RG | Yes | Yes |
| IL3RA.13744.37 | IL3RA | Yes | Yes |
| IL4R.3055.54 | IL4R | Yes | Yes |
| IL5RA.13686.2 | IL5RA | Yes | Yes |
| IL6R.15602.43 | IL6R | Yes | Yes |
| IL7R.5089.11 | IL7R | Yes | Yes |
| IL10RA.8104.21 | IL10RA.soma1 | Yes | Yes |
| IL10RA.10344.334 | IL10RA.soma2 | Yes | Yes |
| IL10RB.2631.50 | IL10RB | Yes | Yes |
| IL11RA.18216.22 | IL11RA | Yes | Yes |
| IL12RB1.2632.5 | IL12RB1 | Yes | Yes |
| IL12RB2.3815.14 | IL12RB2 | Yes | Yes |
| IL13RA1.2633.52 | IL13RA1 | Yes | Yes |
| IL15RA.14054.17 | IL15RA.soma1 | Yes | Yes |
| IL15RA.3445.53 | IL15RA.soma2 | Yes | Yes |
| IL17RA.2992.59 | IL17RA | Yes | Yes |
| IL17RB.6262.14 | IL17RB | Yes | Yes |
| IL17RC.5468.67 | IL17RC | Yes | Yes |
| IL17RD.3376.49 | IL17RD | Yes | Yes |
| IL18R1.14079.14 | IL18R1 | Yes | Yes |
| IL18RAP.2993.1 | IL18RAP | Yes | Yes |
| IL20RA.5085.18 | IL20RA | Yes | Yes |
| IL20RB.13435.31 | IL20RB | Yes | Yes |
| IL21R.9366.54 | IL21R | Yes | Yes |
| IL22RA1.3620.67 | IL22RA1 | Yes | Yes |
| IL22RA2.5087.5 | IL22RA2 | Yes | Yes |
| IL23R.5088.175 | IL23R | Yes | Yes |
| IL27RA.5132.71 | IL27RA | Yes | Yes |
| IL31RA.8273.84 | IL31RA | Yes | Yes |
