# Supplementary material for: The dynamic changes and sex differences of 147 immune-related proteins during acute COVID-19 in 580 individuals
Source: Clin Proteomics. 2022 Sep 28;19:34. doi: 10.1186/s12014-022-09371-z (PMC9516500; doi:10.1186/s12014-022-09371-z)
Supplement: Supplementary file 2 — Additional file 2: Sample code for the generalized additive models. [file 12014_2022_9371_MOESM2_ESM.docx]

**Supplement 2:** sample code for generalized additive models in R^1^, used with the mgcv package^2^.

Where:

1. days_symptom is the number of days from symptoms onset and blood draw
2. case_or_not is a dummy variable (0/1) for whether the patient is a case or a control
3. sex is the sex of the patient
4. age is the age of the patient on enrolment

The gam function options are described in the mgcv manual.

1 R Core Team (2020). R: A language and environment for statistical computing. 2020. https://www.r-project.org/.

2 Wood SN. Generalized Additive Models: An Introduction with R, 2nd edn. Chapman and Hall/CRC, 2017 https://doi.org/10.1201/9781315370279.
