# Supplementary material for: The dynamic changes and sex differences of 147 immune-related proteins during acute COVID-19 in 580 individuals
Source: Clin Proteomics. 2022 Sep 28;19:34. doi: 10.1186/s12014-022-09371-z (PMC9516500; doi:10.1186/s12014-022-09371-z)

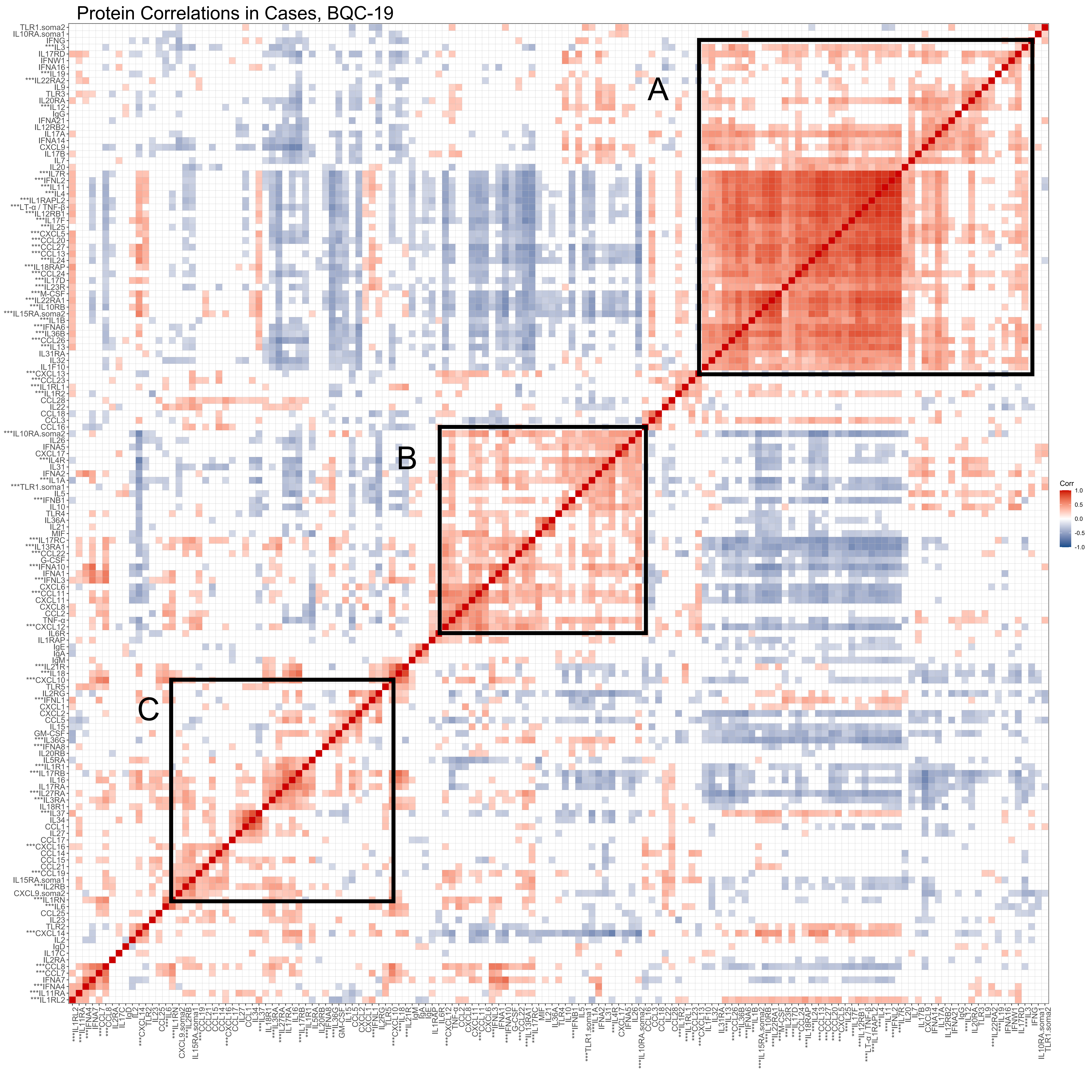
Protein Correlations in Cases, BQC-19
A
B
C
Corr
1.0
0.5
0.0
-0.5
-1.0

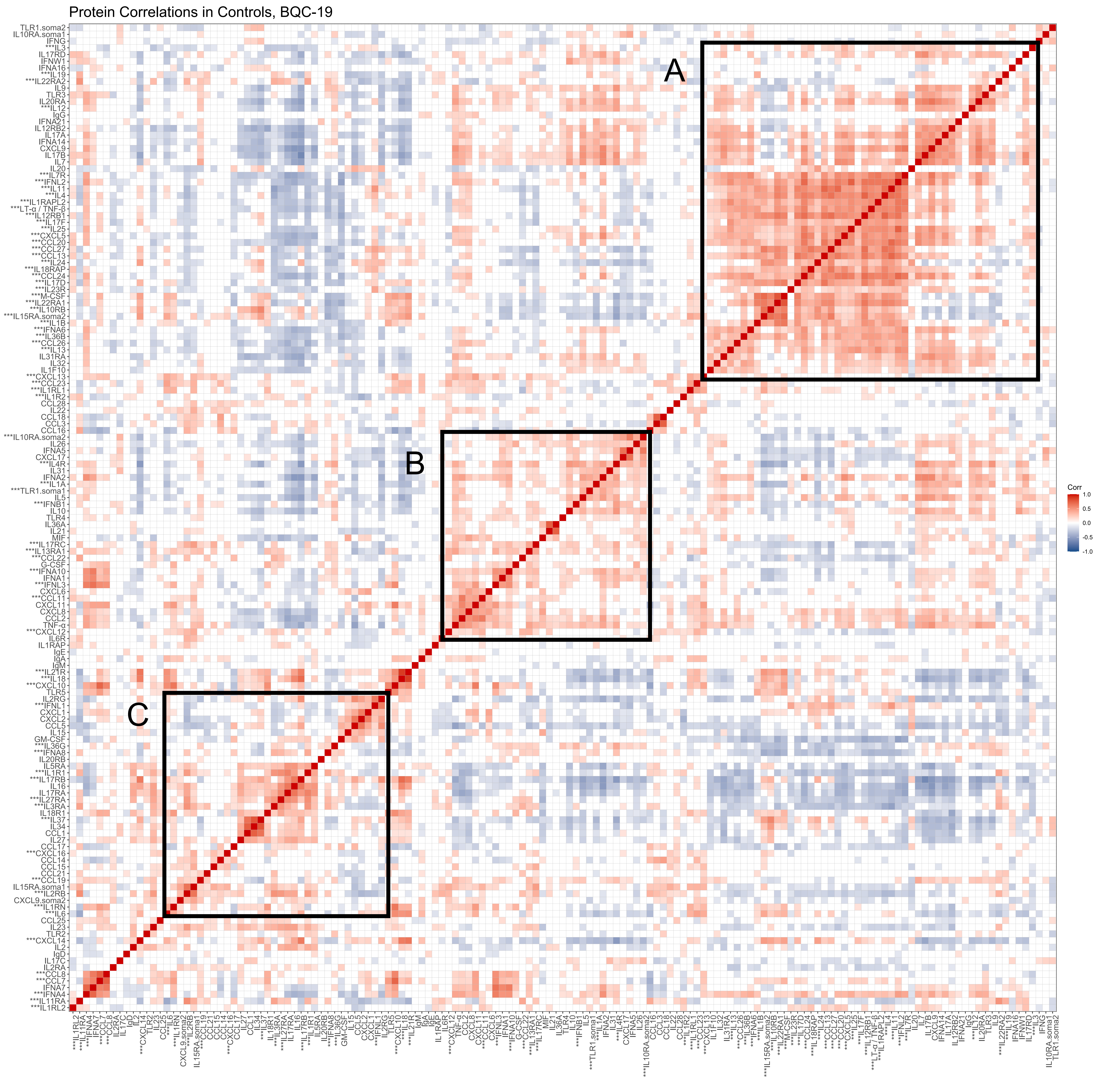

Protein Correlations in Controls, BQC-19
A
B
C
Corr
1.0
0.5
0.0
-0.5
-1.0
TLR1.soma2
IL10RA.soma1
IFNG
***IL3
IL17RD
IFNW1
IFNA16
***IL19
***IL22RA2
IL9
TLR3
IL20RA
***IL12
IgG
IFNA21
IL12RB2
IL17A
IFNA14
CXCL9
IL17B
IL7
IL20
***IL7R
***IFNL2
***IL11
***IL4
***IL1RAPL2
***LT-α / TNF-β
***IL12RB1
***IL17F
***IL25
***CXCL5
***CCL20
***CCL27
***CCL13
***IL24
***IL18RAP
***CCL24
***IL17D
***IL23R
***M-CSF
***IL22RA1
***IL10RB
***IL15RA.soma2
***IL1B
***IFNA6
***IL36B
***CCL26
***IL13
IL31RA
IL32
IL1F10
***CXCL13
***CCL23
***IL1RL1
***IL1R2
CCL28
IL22
CCL18
CCL3
CCL16
***IL10RA.soma2
IL26
IFNA5
CXCL17
***IL4R
IL31
IFNA2
***IL1A
***TLR1.soma1
IL5
***IFNB1
IL10
TLR4
IL36A
IL21
MIF
***IL17RC
***IL13RA1
***CCL22
G-CSF
***IFNA10
IFNA1
***IFNL3
CXCL6
***CCL11
CXCL11
CXCL8
CCL2
TNF-α
***CXCL12
IL6R
IL1RAP
IgE
IgA
IgM
***IL21R
***IL18
***CXCL10
TLR5
IL2RG
***IFNL1
CXCL1
CXCL2
CCL5
IL15
GM-CSF
***IL36G
***IFNA8
IL20RB
IL5RA
***IL1R1
***IL17RB
IL16
IL17RA
***IL27RA
***IL3RA
IL18R1
***IL37
IL34
CCL1
IL27
CCL17
***CXCL16
CCL14
CCL15
CCL21
***CCL19
IL15RA.soma1
***IL2RB
CXCL9.soma2
***IL1RN
***IL6
CCL25
IL23
TLR2
***CXCL14
IL2
IgD
IL17C
IL2RA
***CCL8
***CCL7
IFNA7
***IFNA4
***IL11RA
***IL1RL2

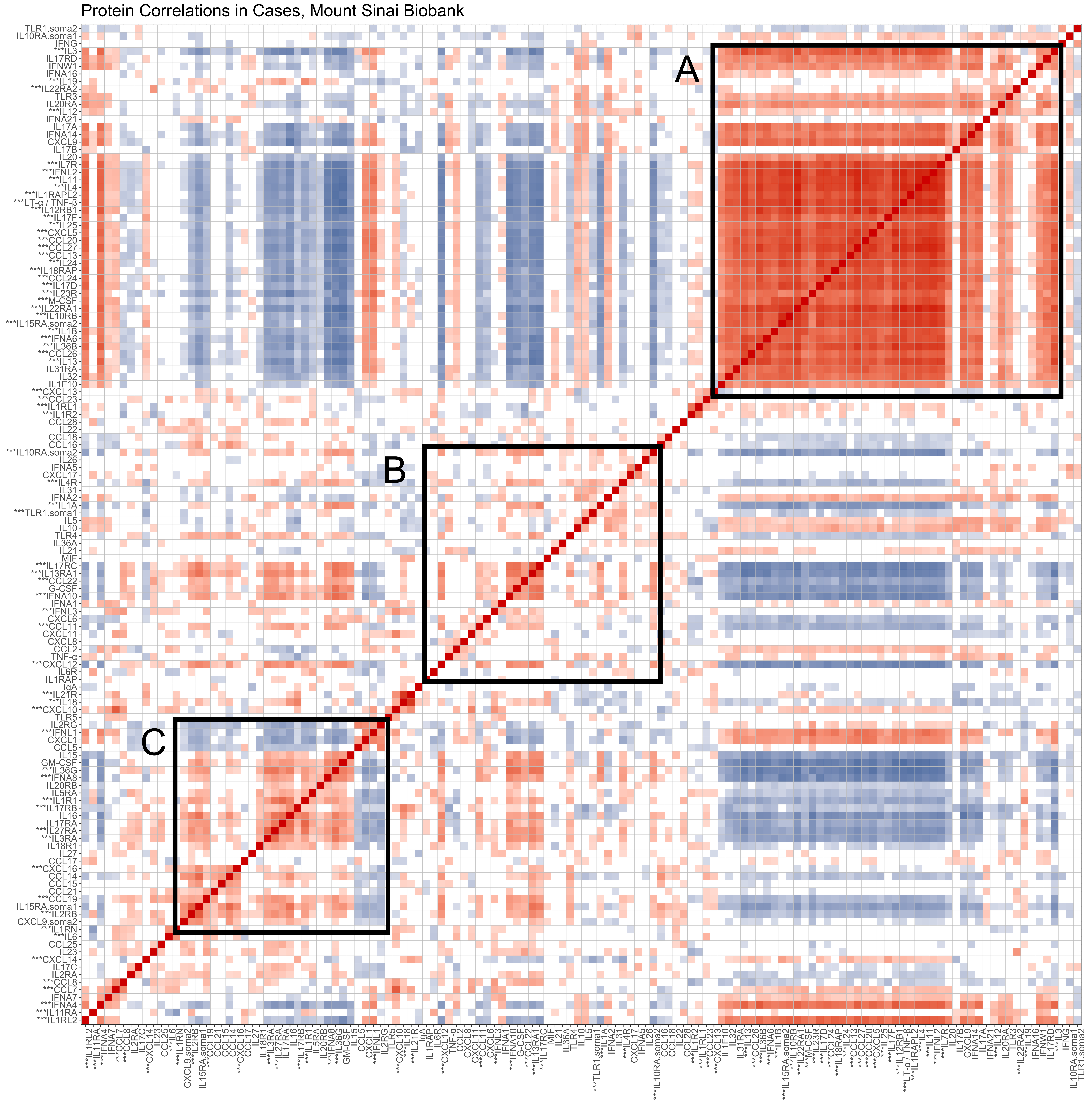
Protein Correlations in Cases, Mount Sinai Biobank
A
B
C
TLR1.soma2
IL10RA.soma1
IFNG
***IL3
IL17RD
IFNW1
IFNA16
***IL19
***IL22RA2
TLR3
IL20RA
***IL12
IFNA21
IL17A
IFNA14
CXCL9
IL17B
IL20
***IL7R
***IFNL2
***IL11
***IL4
***IL1RAPL2
***LT-α / TNF-β
***IL12RB1
***IL17F
***IL25
***CXCL5
***CCL20
***CCL27
***CCL13
***IL24
***IL18RAP
***CCL24
***IL17D
***IL23R
***M-CSF
***IL22RA1
***IL10RB
***IL15RA.soma2
***IL1B
***IFNA6
***IL36B
***CCL26
***IL13
IL31RA
IL32
IL1F10
***CXCL13
***CCL23
***IL1RL1
***IL1R2
CCL28
IL22
CCL18
CCL16
***IL10RA.soma2
IL26
IFNA5
CXCL17
***IL4R
IL31
IFNA2
***IL1A
***TLR1.soma1
IL5
IL10
TLR4
IL36A
IL21
MIF
***IL17RC
***IL13RA1
***CCL22
G-CSF
***IFNA10
IFNA1
***IFNL3
CXCL6
***CCL11
CXCL11
CXCL8
CCL2
TNF-α
***CXCL12
IL6R
IL1RAP
IgA
***IL21R
***IL18
***CXCL10
TLR5
IL2RG
***IFNL1
CXCL1
CCL5
IL15
GM-CSF
***IL36G
***IFNA8
IL20RB
IL5RA
***IL1R1
***IL17RB
IL16
IL17RA
***IL27RA
***IL3RA
IL18R1
IL27
CCL17
***CXCL16
CCL14
CCL15
CCL21
***CCL19
IL15RA.soma1
***IL2RB
CXCL9.soma2
***IL1RN
***IL6
CCL25
IL23
***CXCL14
IL17C
IL2RA
***CCL8
***CCL7
IFNA7
***IFNA4
***IL11RA
***IL1RL2

Protein Correlations in Controls, Mount Sinai Biobank

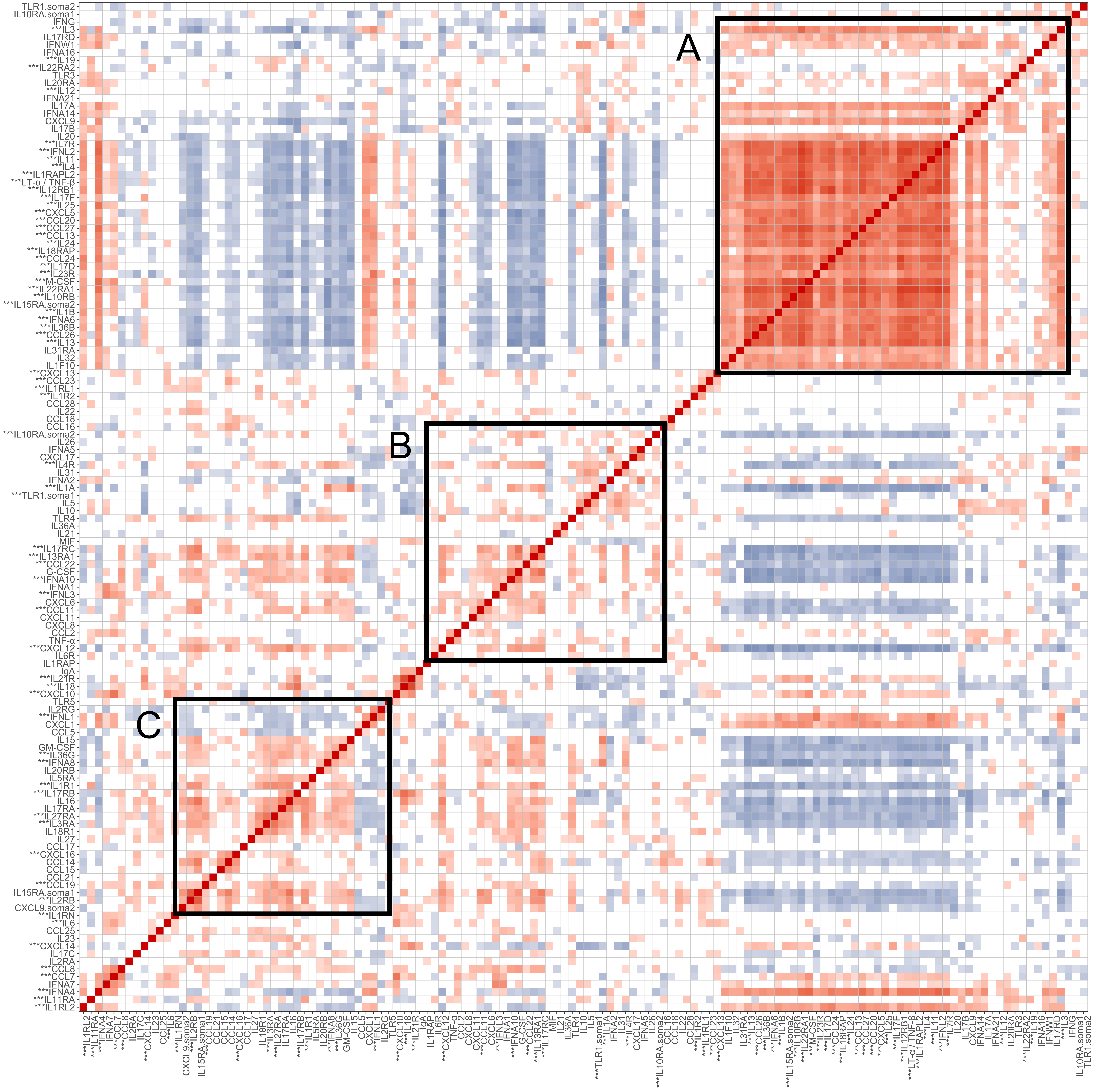

Supplement: Supplementary file 3 — Additional file 3: Protein correlation heatmaps. [file 12014_2022_9371_MOESM3_ESM.pdf]
