# Supplementary material for: The dynamic changes and sex differences of 147 immune-related proteins during acute COVID-19 in 580 individuals
Source: Clin Proteomics. 2022 Sep 28;19:34. doi: 10.1186/s12014-022-09371-z (PMC9516500; doi:10.1186/s12014-022-09371-z)
Supplement: Supplementary file 4 — Additional file 4: Protein clusters. [file 12014_2022_9371_MOESM4_ESM.docx]

| **Cluster A** | **Cluster B** | **Cluster C** |
| --- | --- | --- |
| ***IL3 | ***IL10RA.soma2 | IL2RG |
| IL17RD | IL26 | ***IFNL1 |
| IFNW1 | IFNA5 | CXCL1 |
| IFNA16 | CXCL17 | CXCL2 |
| ***IL19 | ***IL4R | CCL5 |
| ***IL22RA2 | IL31 | IL15 |
| IL9 | IFNA2 | GM-CSF |
| TLR3 | ***IL1A | ***IL36G |
| IL20RA | ***TLR1.soma1 | ***IFNA8 |
| ***IL12 | IL5 | IL20RB |
| IgG | ***IFNB1 | IL5RA |
| IFNA21 | IL10 | ***IL1R1 |
| IL12RB2 | TLR4 | ***IL17RB |
| IL17A | IL36A | IL16 |
| IFNA14 | IL21 | IL17RA |
| CXCL9 | MIF | ***IL27RA |
| IL17B | ***IL17RC | ***IL3RA |
| IL7 | ***IL13RA1 | IL18R1 |
| IL20 | ***CCL22 | ***IL37 |
| ***IL7R | G-CSF | IL34 |
| ***IFNL2 | ***IFNA10 | CCL1 |
| ***IL11 | IFNA1 | IL27 |
| ***IL4 | ***IFNL3 | CCL17 |
| ***IL1RAPL2 | CXCL6 | ***CXCL16 |
| ***LT-α / TNF-β | ***CCL11 | CCL14 |
| ***IL12RB1 | CXCL11 | CCL15 |
| ***IL17F | CXCL8 | CCL21 |
| ***IL25 | CCL2 |  |
| ***CXCL5 | TNF-α |  |
| ***CCL20 | ***CXCL12 |  |
| ***CCL27 | ***IL10RA.soma2 |  |
| ***CCL13 | IL26 |  |
| ***IL24 |  |  |
| ***IL18RAP |  |  |
| ***CCL24 |  |  |
| ***IL17D |  |  |
| ***IL23R |  |  |
| ***M-CSF |  |  |
| ***IL22RA1 |  |  |
| ***IL10RB |  |  |
| ***IL15RA.soma2 |  |  |
| ***IL1B |  |  |
| ***IFNA6 |  |  |
| ***IL36B |  |  |
| ***CCL26 |  |  |
| ***IL13 |  |  |
| IL31RA |  |  |
| IL32 |  |  |
| IL1F10 |  |  |

**Supplement 4**: Cytokines and related immune proteins in each of the three clusters identified by hierarchical clustering algorithm (HCA) and spearman correlation. Proteins with preceded by asterisks (***) were found to show a statistically significant time-dependent difference between severe Covid-19 cases and controls. Proteins ordered as per their HCA ordering.
