# Supplementary material for: The dynamic changes and sex differences of 147 immune-related proteins during acute COVID-19 in 580 individuals
Source: Clin Proteomics. 2022 Sep 28;19:34. doi: 10.1186/s12014-022-09371-z (PMC9516500; doi:10.1186/s12014-022-09371-z)

**Supplement 5:** protein level trends and 95% confidence intervals for severe Covid-19 cases and controls, using generalized additive model inference and cubic splines for the first 14 days of since onset of symptoms (x-axes). These are presented for 65-year-old females and males. For 15 proteins, no measurement was available in the Mount Sinai Biobank, and the results shown are from the BQC19 only. These are indicated on the relevant plots.

**Interleukins:**


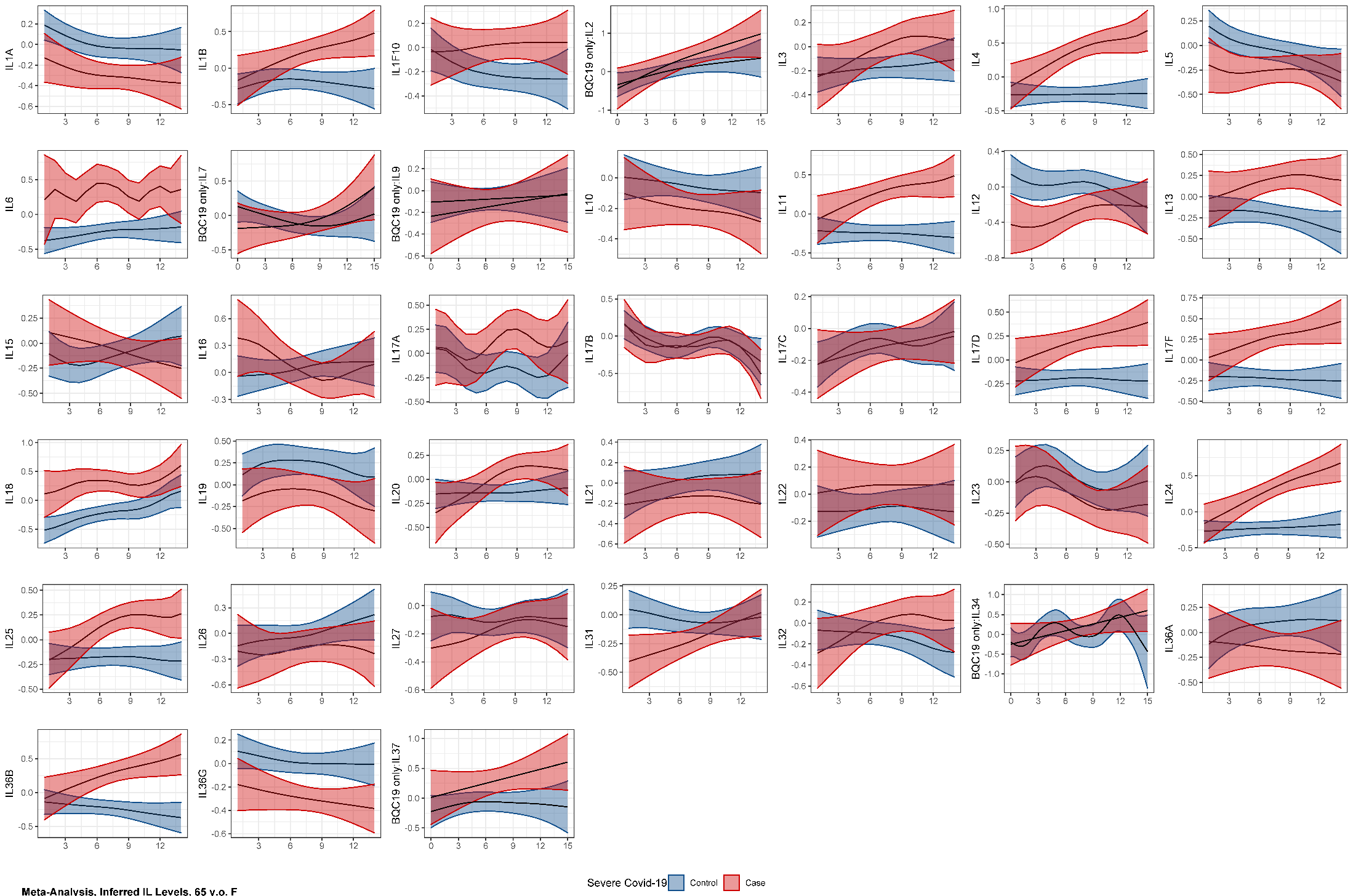


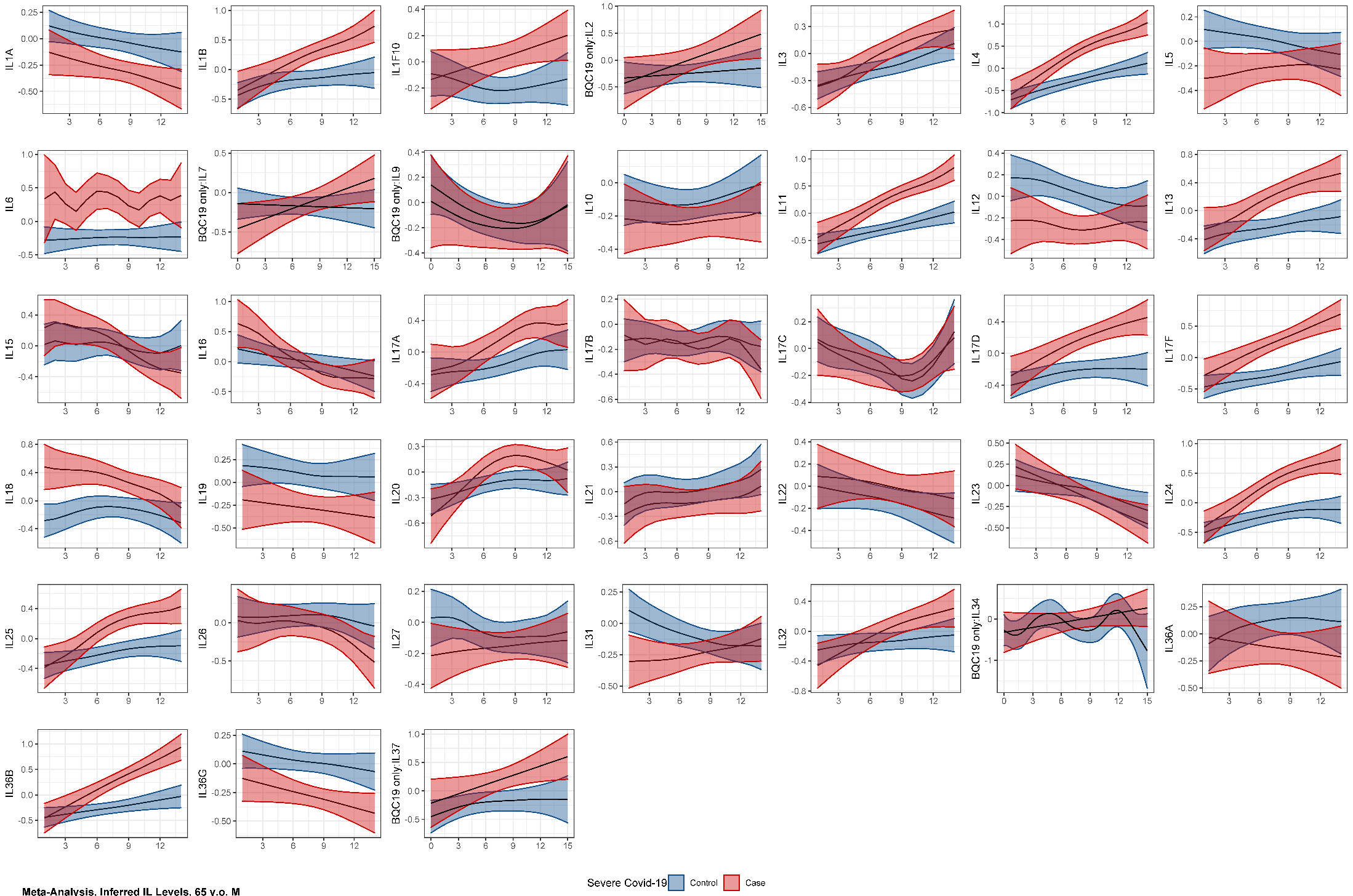


**Soluble interleukin receptors:**


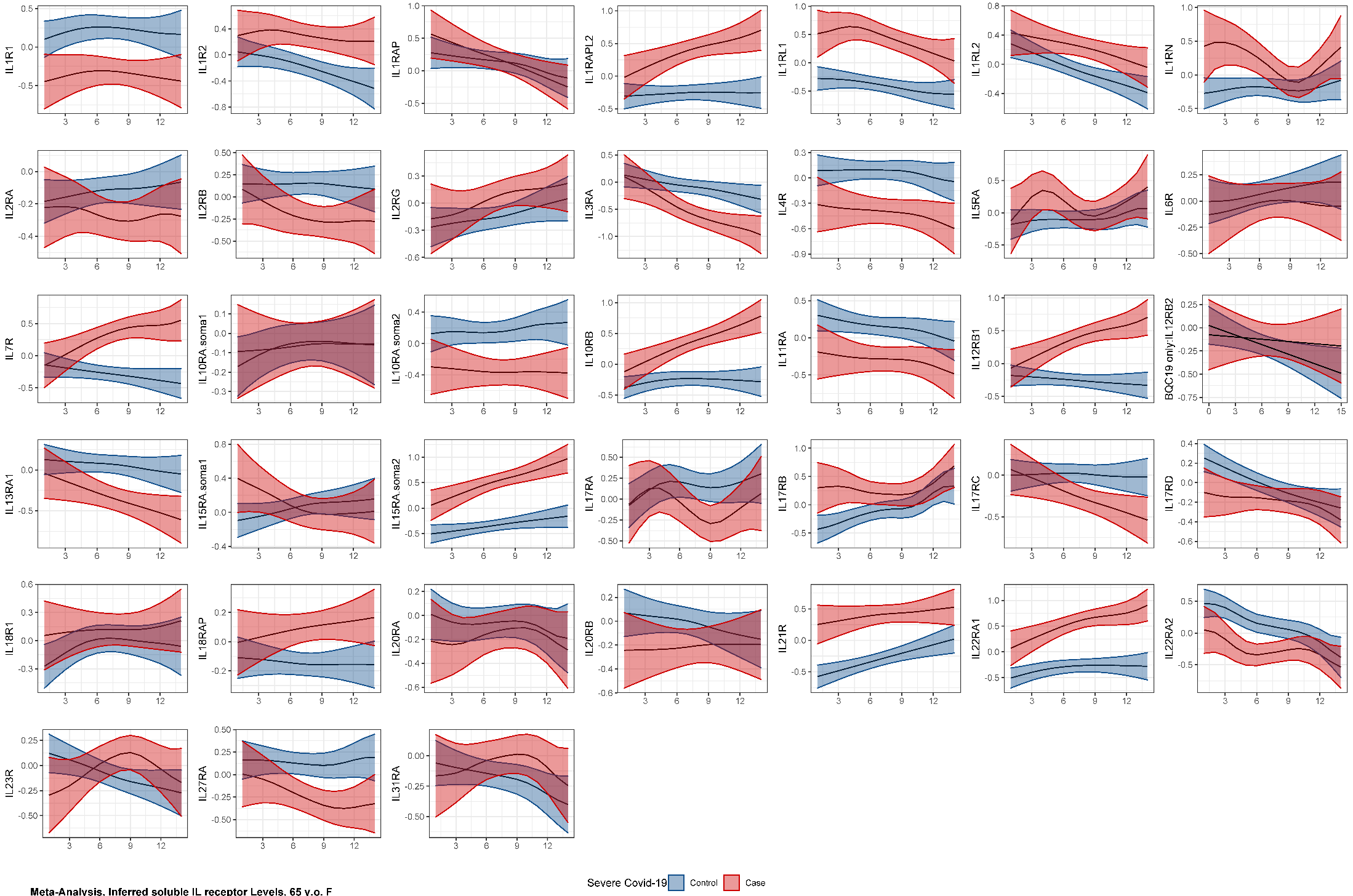


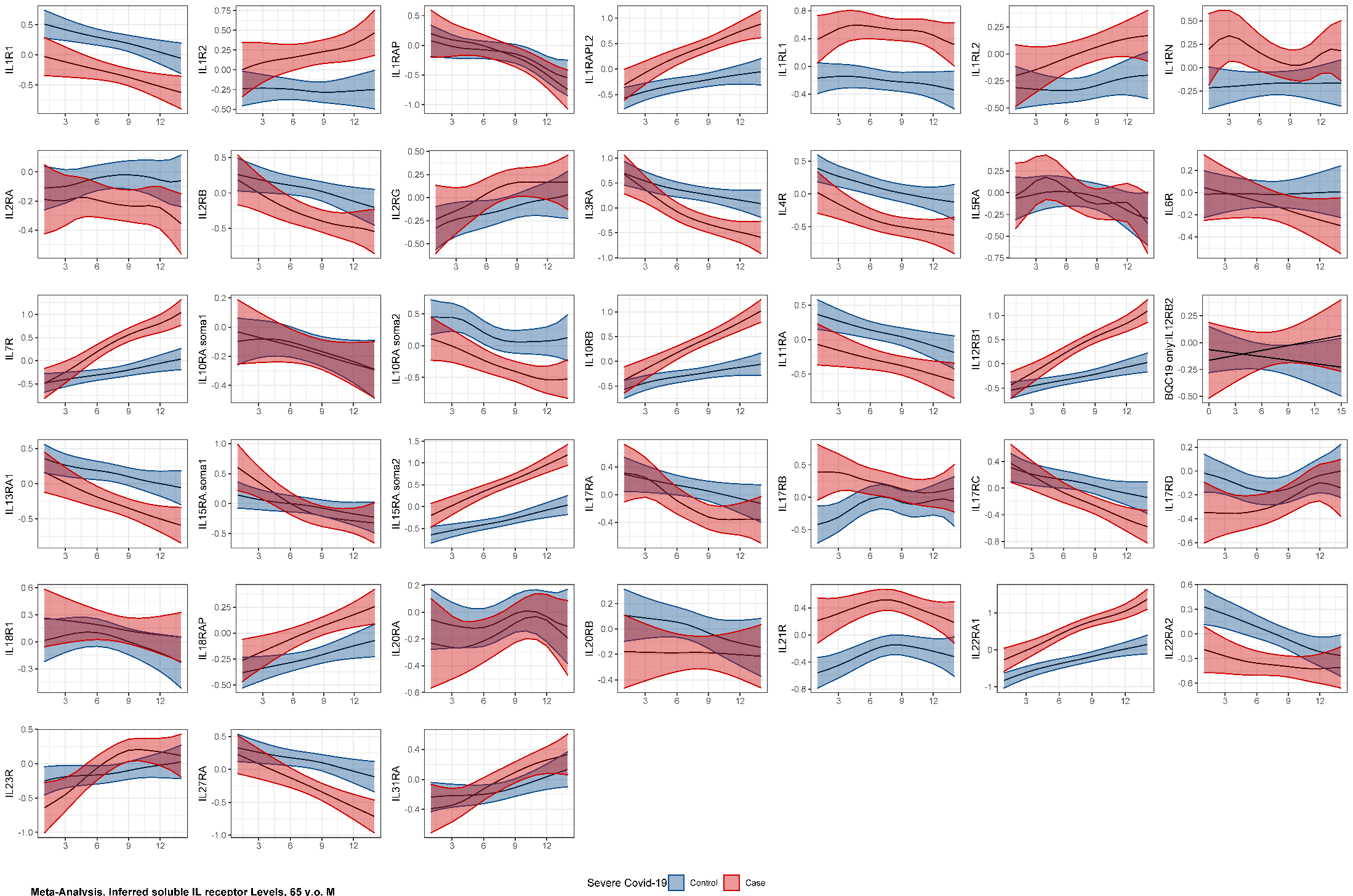


**CC chemokines:**


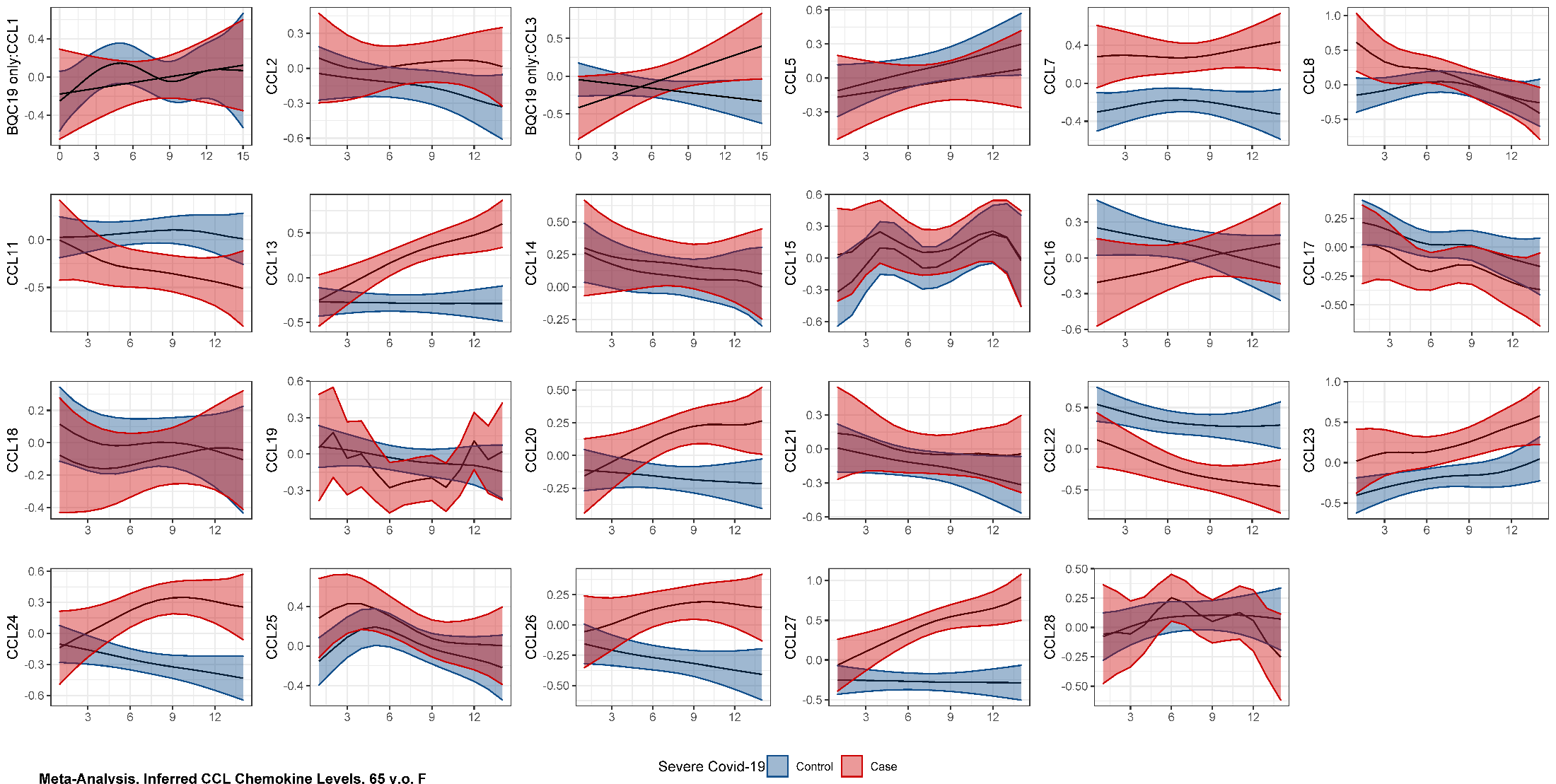


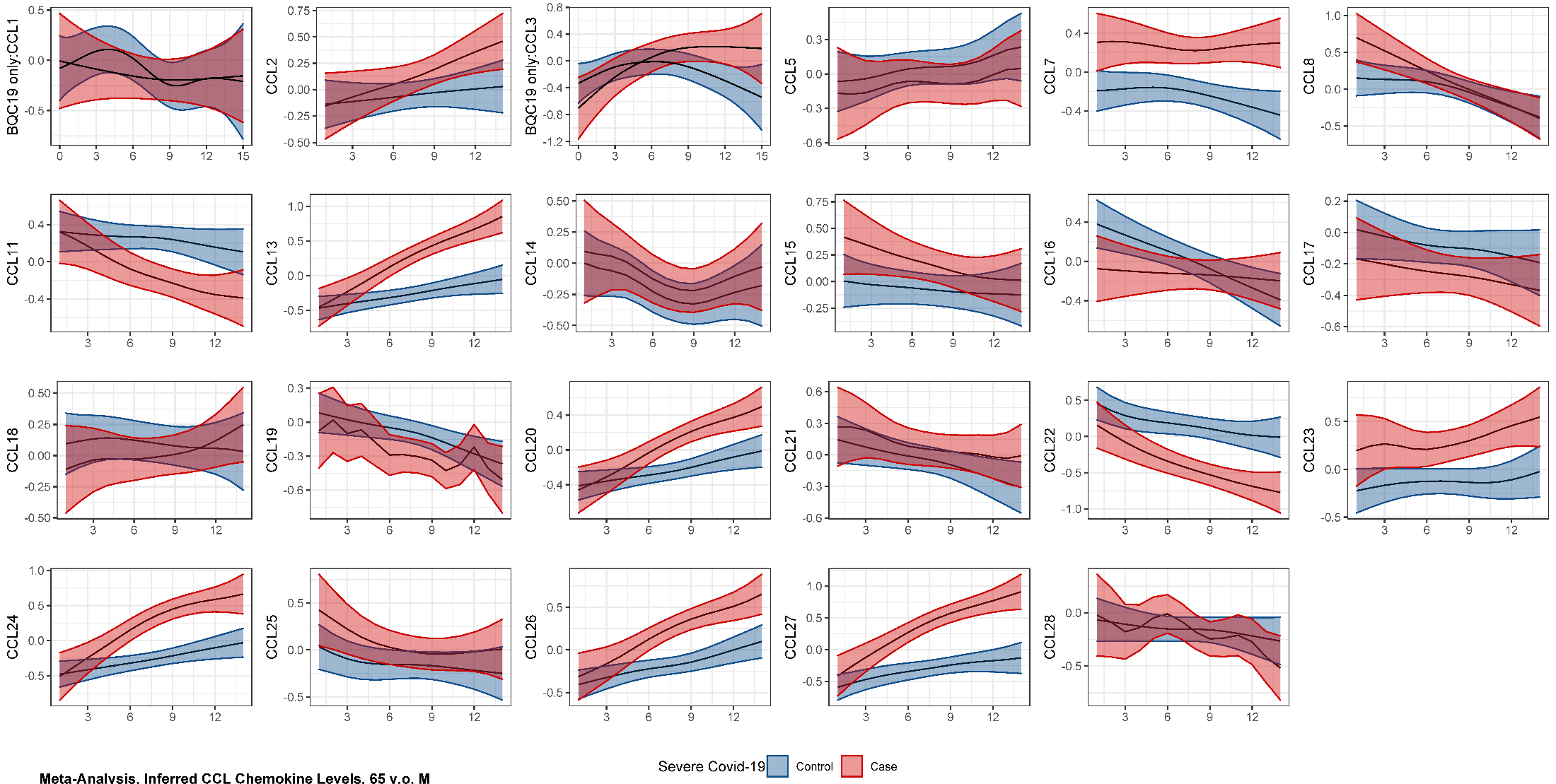


**CXC chemokines:**

**
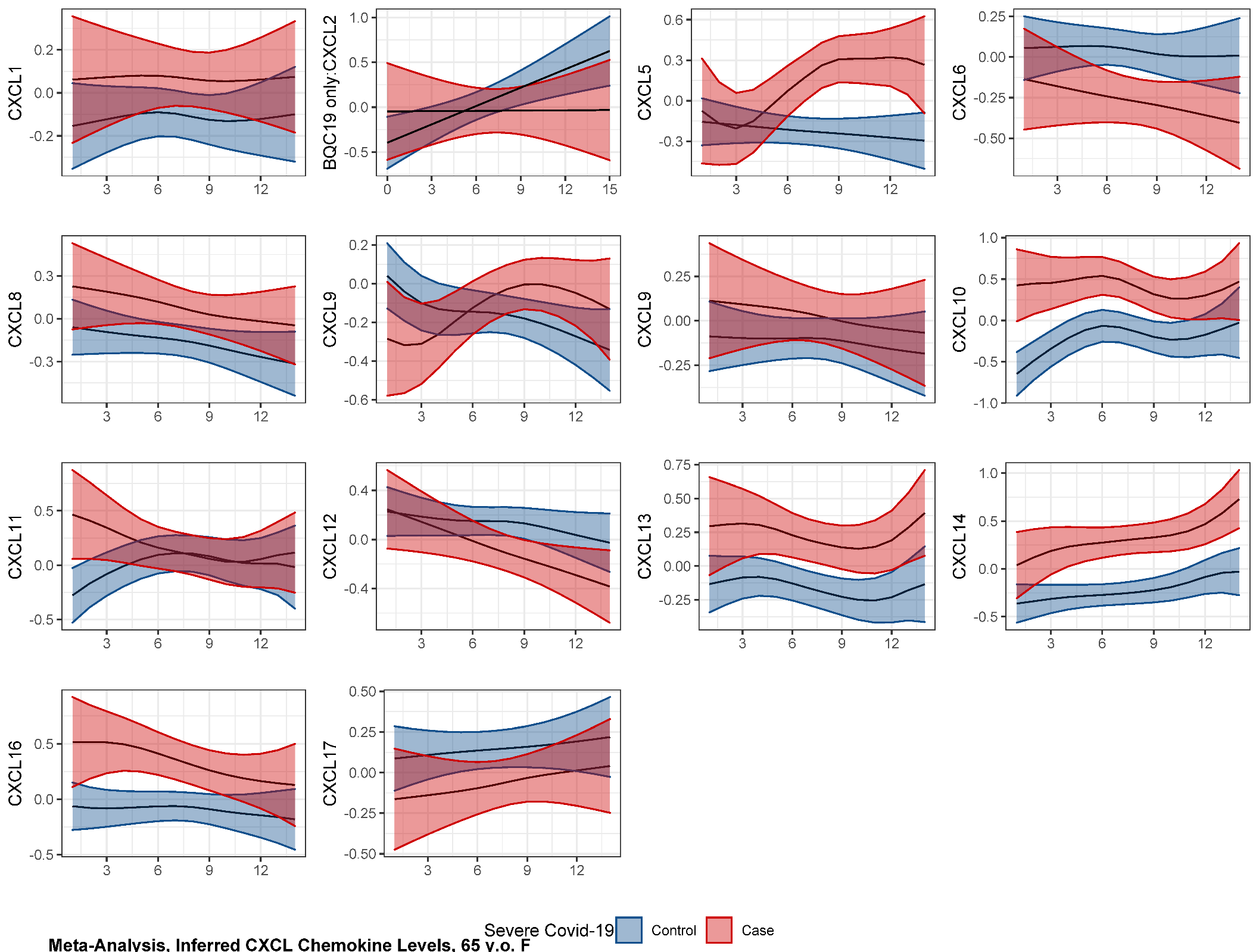
**


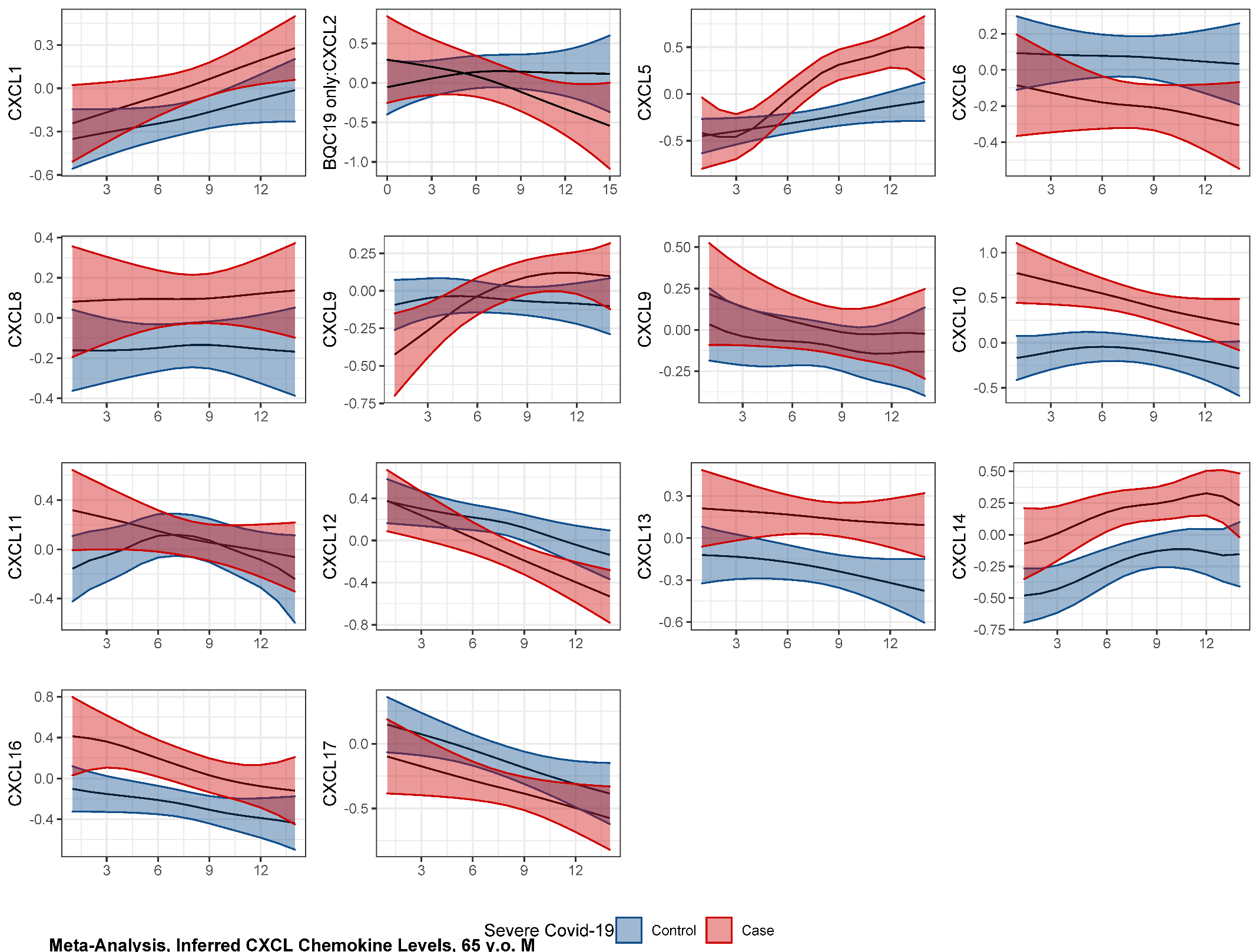


**Interferons:**


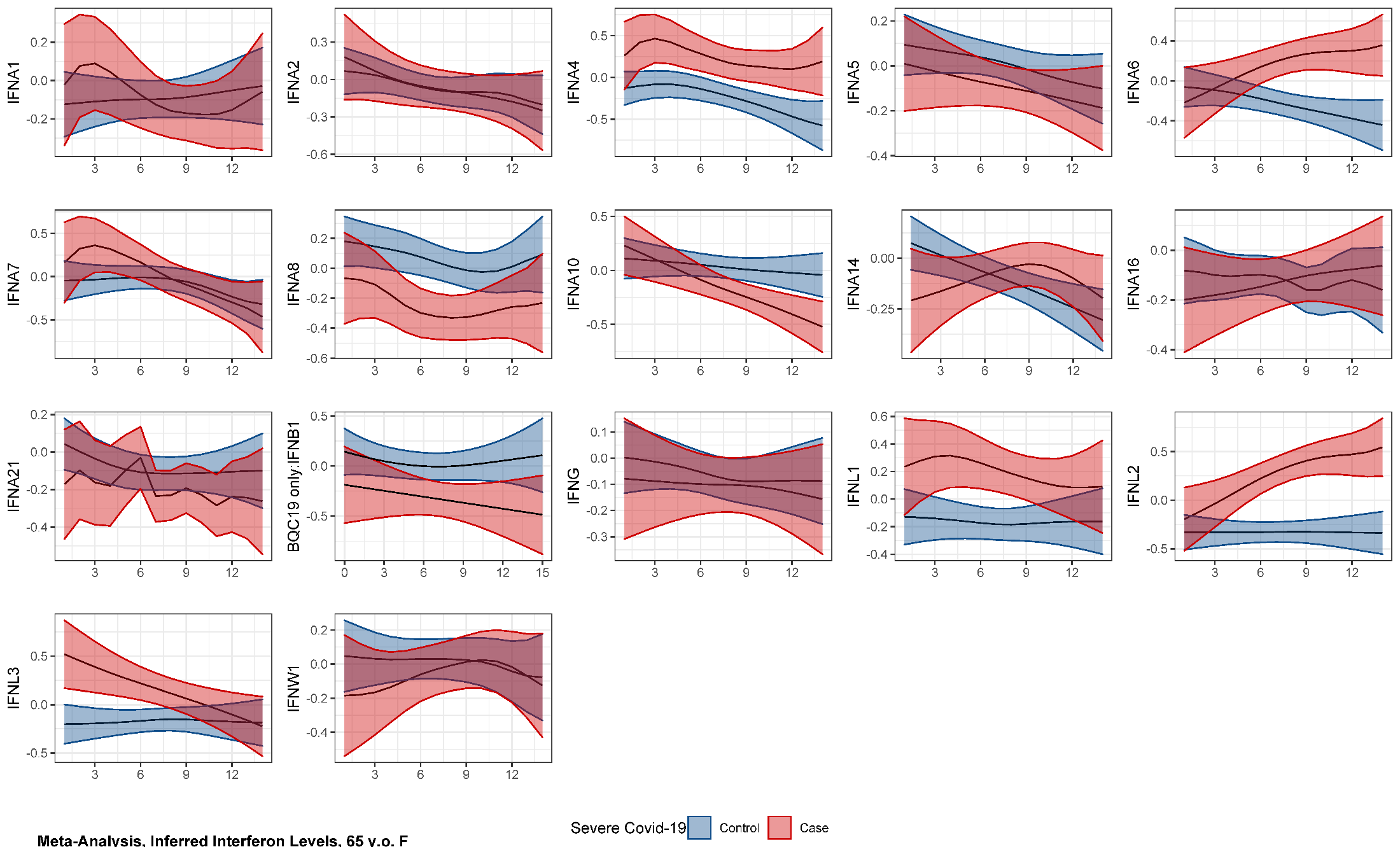


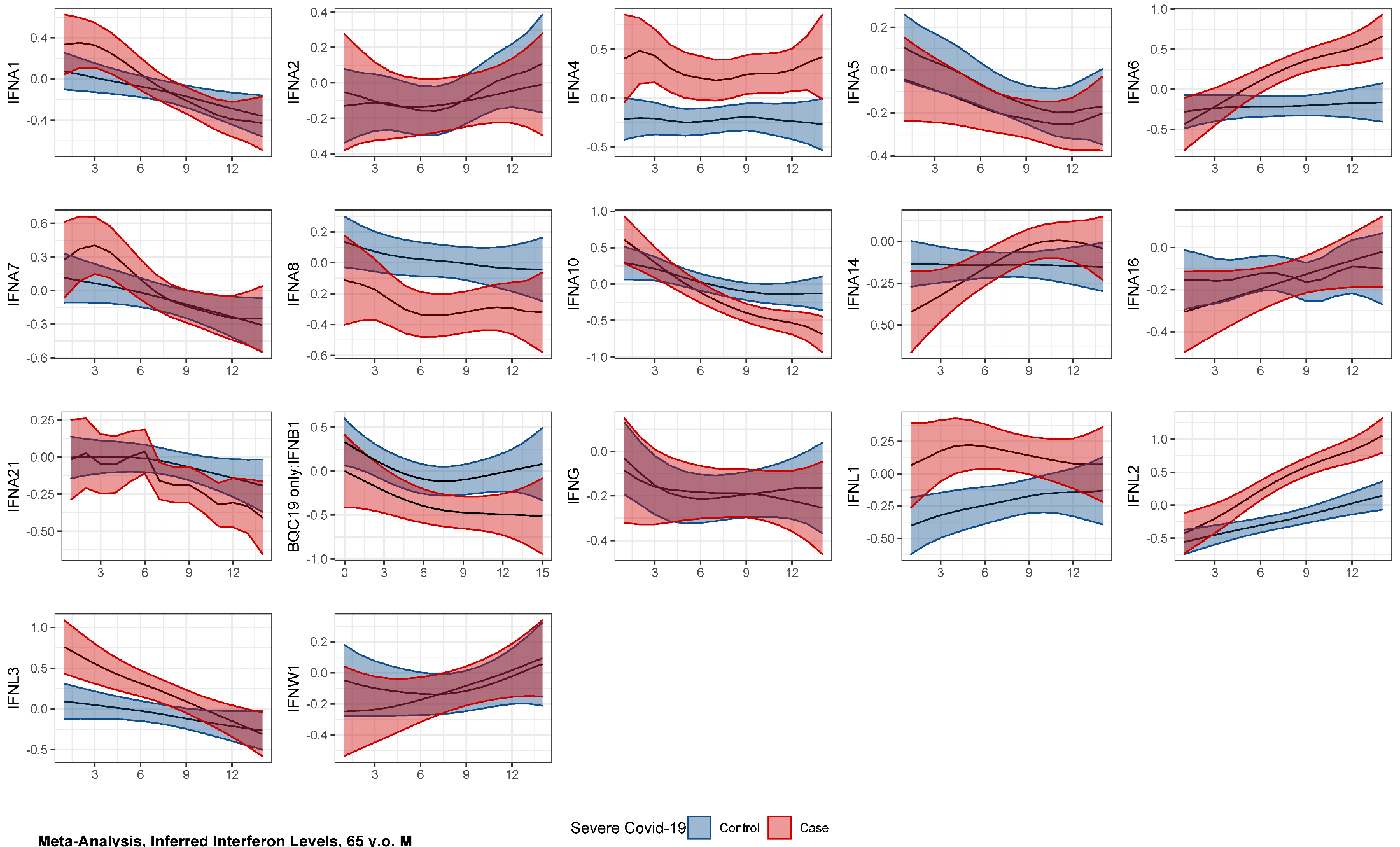


**Other proteins:**


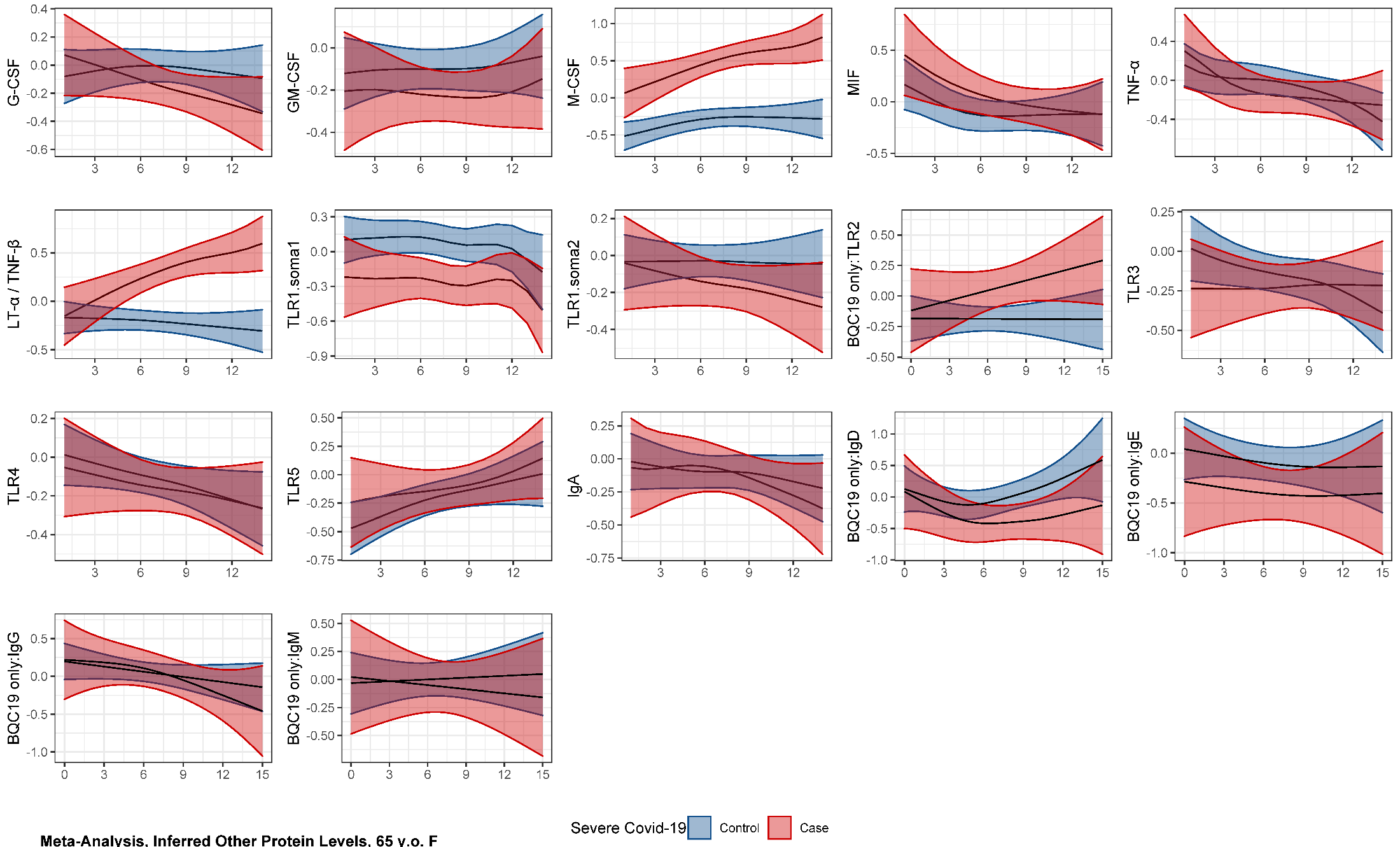


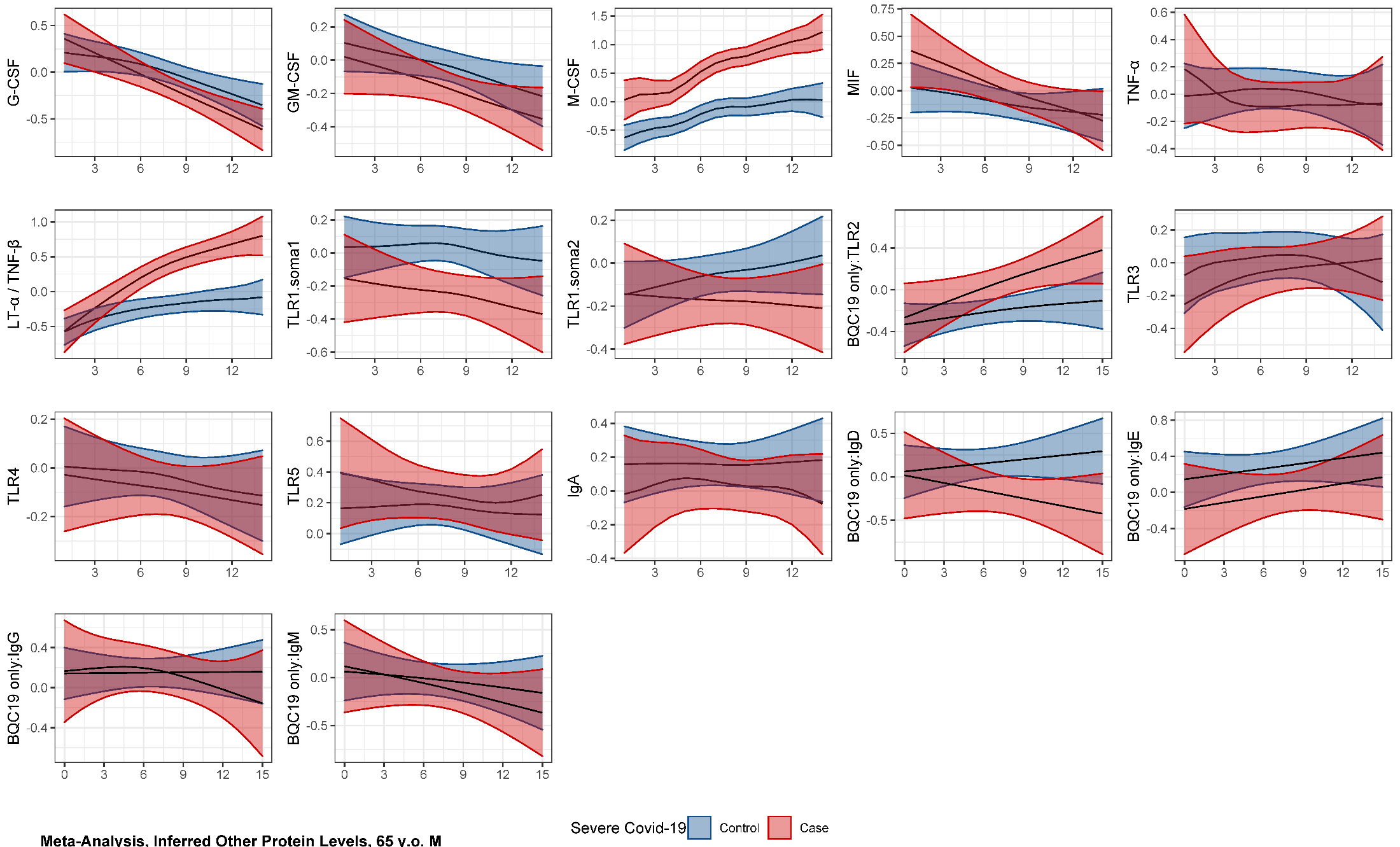

Supplement: Supplementary file 5 — Additional file 5: Inferred protein levels over time. [file 12014_2022_9371_MOESM5_ESM.docx]
