# Supplementary material for: The dynamic changes and sex differences of 147 immune-related proteins during acute COVID-19 in 580 individuals
Source: Clin Proteomics. 2022 Sep 28;19:34. doi: 10.1186/s12014-022-09371-z (PMC9516500; doi:10.1186/s12014-022-09371-z)
Supplement: Supplementary file 6 — Additional file 6: Mount Sinai investigators. [file 12014_2022_9371_MOESM6_ESM.docx]

**Supplement 6**: Members of the Mount Sinai COVID-19 Biobank Team

Charuta Agashe

Priyal Agrawal

Alara Akyatan

Kasey Alesso-Carra

Eziwoma Alibo

Kelvin Alvarez

Angelo Amabile

Carmen Argmann

Kimberly Argueta

Steven Ascolillo

Rasheed Bailey

Craig Batchelor

Noam D. Beckmann

Aviva G. Beckmann

Priya Begani

Jessica Le Berichel

Dusan Bogunovic

Swaroop Bose

Cansu Cimen Bozkus

Paloma Bravo

Mark Buckup

Larissa Burka

Sharlene Calorossi

Lena Cambron

Guillermo Carbonell

Gina Carrara

Mario A. Cedillo

Christie Chang

Serena Chang

Alexander W. Charney

Steven T. Chen

Esther Cheng

Jonathan Chien

Mashkura Chowdhury

Jonathan Chung

Phillip H. Comella

Dana Cosgrove

Francesca Cossarini

Liam Cotter

Arpit Dave

Travis Dawson

Bheesham Dayal

Diane Marie Del Valle

Maxime Dhainaut

Rebecca Dornfeld

Katie Dul

Melody Eaton

Nissan Eber

Cordelia Elaiho

Ethan Ellis

Frank Fabris

Jeremiah Faith

Dominique Falci

Susie Feng

Brian Fennessy

Marie Fernandes

Nataly Fishman

Nancy J. Francoeur

Sandeep Gangadharan

Daniel Geanon

Bruce D. Gelb

Benjamin S. Glicksberg

Sacha Gnjatic

Joanna Grabowska

Gavin Gyimesi

Maha Hamdani

Diana Handler

Jocelyn Harris

Matthew Hartnett

Sandra Hatem

Manon Herbinet

Elva Herrera

Arielle Hochman

Gabriel E. Hoffman

Jaime Hook

Laila Horta

Etienne Humblin

Suraj Jaladanki

Hajra Jamal

Jessica S. Johnson

Gurpawan Kang

Neha Karekar

Subha Karim

Geoffrey Kelly

Jong Kim

Seunghee Kim-Schulze

Edgar Kozlova

Arvind Kumar

Jose Lacunza

Alona Lansky

Dannielle Lebovitch

Brian Lee

Grace Lee

Gyu Ho Lee

Jacky Lee

John Leech

Lauren Lepow

Michael B. Leventhal

Lora E. Liharska

Katherine Lindblad

Alexandra Livanos

Bojan Losic

Rosalie Machado

Kent Madrid

Zafar Mahmood

Kelcey Mar

Thomas U. Marron

Glenn Martin

Robert Marvin

Shrisha Maskey

Paul Matthews

Katherine Meckel

Saurabh Mehandru

Miriam Merad

Cynthia Mercedes

Elyze Merzier

Dara Meyer

Gurkan Mollaoglu

Sarah Morris

Konstantinos Mouskas

Emily Moya

Naa-akomaah Yeboah

Girish Nadkarni

Kai Nie

Marjorie Nisenholtz

George Ofori-Amanfo

Kenan Onel

Merouane Ounadjela

Manishkumar Patel

Vishwendra Patel

Cassandra Pruitt

Adeeb Rahman

Shivani Rathi

Jamie Redes

Ivan Reyes-Torres

Alcina Rodrigues

Alfonso Rodriguez

Vladimir Roudko

Panagiotis Roussos

Evelyn Ruiz

Pearl Scalzo

Eric E. Schadt

Ieisha Scott

Robert Sebra

Hardik Shah

Mark Shervey

Pedro Silva

Nicole W. Simons

Melissa Smith

Alessandra Soares Schanoski

Juan Soto

Shwetha Hara Sridhar

Stacey-Ann Brown

Hiyab Stefanos

Meghan Straw

Robert Sweeney

Alexandra Tabachnikova

Collin Teague

Ryan Thompson

Manying Tin

Kevin Tuballes

Scott R. Tyler

Bhaskar Upadhyaya

Akhil Vaid

Verena Van Der Heide

Natalie Vaninov

Konstantinos Vlachos

Daniel Wacker

Laura Walker

Hadley Walsh

Wenhui Wang

Bo Wang

C. Matthias Wilk

Lillian Wilkins

Karen M. Wilson

Jessica Wilson

Hui Xie

Li Xue

Nancy Yi

Ying-chih Wang

Mahlet Yishak

Sabina Young

Alex Yu

Nina Zaks

Renyuan Zha
